# Utilization of cell-penetrating peptide adaptors to enhance delivery of variably charged protein cargos

**DOI:** 10.1371/journal.pone.0345530

**Published:** 2026-07-10

**Authors:** Daniel P. Morris, Nathaniel I. Turner, Jojo J. Croffie, Jonathan L. McMurry

**Affiliations:** Department of Molecular & Cellular Biology, Kennesaw State University, Kennesaw, Georgia, United States of America; Max-Planck-Institut fur Polymerforschung, GERMANY

## Abstract

Cell-penetrating peptides (CPPs) can deliver biomacromolecular cargos into cells, potentially enabling a new mode of intracellular drug delivery. However, a major problem with CPP-mediated delivery is entrapment of CPPs within endosomes as covalent linkages ensure CPPs and cargos share common fates. We previously developed a CPP-adaptor system based on reversible, calcium-dependent cargo binding that produces cargo release from adaptors as complexes dissociate following internalization and Ca^2+^ efflux from early endosomes. Having employed CPP-adaptors with an array of protein cargos of differing charges, it became apparent that positively charged cargos often appeared to dominate internalization and that association with the adaptor had little effect. To systematically address the effects of cargo charge and CPP function, we tested the ability of several adaptors to increase internalization of a set of adaptor binding GFP cargos having charges of +9, + 15, + 20, + 25 and +36. Intrinsic internalization of these cargos reproduced reported patterns showing that positive charge increases internalization. Interestingly, labeling these cargos with a chemical fluorophore revealed that GFP fluorescence grossly underestimated total internalization as shown by the fluor. Internalization was charge and concentration dependent with more positive cargos showing apparent saturation of internalization at 100–400 nM, well below the concentrations at which covalently linked CPP-cargos are commonly dosed. We tested the ability of 5 adaptors to internalize these cargos. Our prototype adaptor, TAT-CaM, was completely ineffective with the + 9 cargo, but internalized moderately charged cargos extremely efficiently at concentrations far below the µM range. A derivative adaptor, TAT-LAH4-CaM, was highly effective with all cargos and produced similar maximal internalization at 100–400 nM. However, two adaptors specifically designed with increased positive charge inhibited internalization of the most positive cargos. One of these, GFP-CaM, based on the supercharged GFP with net charge of +36, did increase internalization of the least positive cargos, demonstrating an adaptor with high affinity for the cell surface can increase internalization of a neutral cargo at very low concentration. The common maximal level of intrinsic GFP cargo internalization correlated with surface loading of these cargos, suggesting a limit to the beneficial effects of increased plasma membrane association. However, TAT-CaM further increased internalization above intrinsic levels via an apparently distinct mechanism. In this limited study of the interaction of cargo charge and adaptor efficacy, we found diverse behaviors that hint at the power and flexibility possible with adaptor/cargo internalization.

## Introduction

Inability to cross membranes is a common failure of otherwise promising therapeutic leads. Cell-penetrating peptides (CPPs) have long held promise for overcoming these failures by efficient, nonviral transmembrane delivery of biomolecular “cargos” to the cytoplasm or other subcellular destination wherein their therapeutic targets are located. Classically, a CPP is covalently attached to a protein or other biomolecule. The CPP binds to cell surface proteoglycan receptors, triggering endocytosis and bringing the cargo along with it [1]. However, it has long been recognized that cargo delivery to the cytoplasm is very inefficient because CPP-cargos become trapped in endosomes and are either recycled to the cell surface or targeted for degradation. This is perhaps the major reason that no CPP-based drug has yet been approved by the U.S. FDA despite dozens of clinical trials [2].

To overcome this endosomal escape problem, we developed CPP-adaptors that spontaneously form noncovalent complexes with cargos via Ca^2+^ dependent coupling. Our prototype, TAT-CaM, consists of the well-characterized CPP TAT [3,4] N-terminally fused to calmodulin [5]. Other adaptors have different CPPs, different EF hand proteins, and/or additional features such as endosomal escape enhancing peptide (also called endolytic peptide, “EP”) sequences [6,7]. CPP-adaptors bind calmodulin binding sequence (CBS) containing cargo proteins with low nM affinity in the presence of Ca^2+^ and negligibly in its absence [5,8]. Thus, CPP-adaptor/CBS-cargo complexes bind tightly in extracellular media and during internalization but dissociate after formation of endosomes as Ca^2+^ efflux from endosomes occurs during trafficking [9], leaving cargo to egress to the cytoplasm while the adaptors remain trapped like other CPPs [6,7,10,11]. While dissociation enhanced cargo escape remains a critical adaptor function, our use of CPP-adaptors also suggested that separation of cargo and adaptor function provided a powerful model for investigation of CPP-mediated delivery.

It is well established that positive charge is part of the mechanism used by CPPs to gain cell entry [12]. Evidence suggests this is due to association of positive CPPs with negatively charged proteoglycans on the cell surface [13,14]. Very recently, an unbiased genetic screen used to identify target cell proteins that aid internalization of TAT-associated cargos identified proteins responsible for synthesis of proteoglycans, providing very strong support for this idea [15]. It has also been recognized that “supercharged” green fluorescent proteins (GFPs) [16,17], in which surface residues have been altered to increase positive surface charge, act as cell-penetrating proteins (CPPrs) with modest intrinsic internalization [18]. This points to the obvious fact that CPPs fused to cargo proteins display internalization properties clearly dependent on both the CPP and cargo charge, greatly confusing determination of CPP efficacy. The interaction of CPP function and intrinsic cargo internalization due to positive charge has not been systematically investigated.

Because our CPP-adaptors allow separation of intrinsic cargo internalization from the effects of CPP-adaptor/cargo complexes, the interaction of cargo charge and CPP-adaptor function can easily be compared. Based on the supercharged GFPs mentioned above, we created sets of cargos with CBS-GFPs having net charges of 9, 15, 20, 25 and 36. Using confocal microscopy we investigated internalization of these cargos alone and in complex with a set of 5 adaptors previously shown to be efficacious [7].

Two improved adaptors used an increase in net positive charge to mitigate the negative effects on internalization of the strongly negative calmodulin. One of these, TAT-NMR-CaM, was based on *Heterocephalus glaber* (naked mole rat) calmodulin, which includes an additional positive domain amino terminal to the conserved calmodulin core sequence [10]. The second, GFP-CaM, dispensed with the TAT sequence and used the supercharged GFP with a net + 36 as the sole CPPr [7].

The other two adaptors were initially designed to increase endosomal escape by incorporating putative EP sequences between TAT and CaM. The LAH4 sequence in TAT-LAH4-CaM dramatically increased internalization of cargos based on maltose binding protein, which has a net negative charge [10]. LAH4 was identified as an EP and numerous reports have since shown it has CPP functionality [19,20]. The second adaptor, TAT-AUR-CaM, incorporated the Aurein 1.2 sequence which has endolytic properties in the BHK cell line used herein [21].

Comparing internalization of supercharged GFP cargos with differing net positive charges produced outcomes that were cargo charge- and concentration-dependent. A maximal level of internalization was associated with apparent saturation of surface binding, suggesting the major factor necessary for internalization can be positive charge. On the other hand, TAT-CaM and TAT-LAH4-CaM further increased internalization suggesting an additional mechanism of action.

## Materials & methods

### Plasmids

Plasmids used were previously described [7] or constructed as described in Supporting Information (S1 Fig). *E. coli-*optimized synthetic genes were designed, synthesized and cloned (GeneScript, Piscataway, NJ, USA) into NdeI or NcoI and BamHI sites in pET19b (EMD Millipore, USA) with an in-frame stop codon prior to the BamHI site. In the case of CPP-adaptors, the N-terminal His tag was followed by the TAT peptide sequence (YGRKKRRQRRR). GFP-CaM consists of sequence encoding the + 36 GFP described by McNaughton *et al* [17] fused to human calmodulin cloned into the NcoI and BamHI wherein the synthetic gene possesses a 6xHis tag after an initiating Met-Gly encoded by the NcoI site.

Cargo protein genes were based on the series of supercharged enhanced GFPs described by Thompson *et al* [16] fused to a canonical calmodulin binding site (KRRWKKNFIAVSAANRFKKISSSGAL) and HiBiT sequence (VSGWRLFKKISGGSG) cloned into NdeI and BamHI sites so that the N-terminal His tag is followed by GFP then CBS then HiBiT in constructs denoted “GCH” or CBS then GFP then HiBiT in constructs denoted “CGH.” Rearrangement of the order of moieties in the fusion was necessary for reasons described below. In no case were His tags removed. CBS and HiBiT sequences contributed to net protein charge. Proteins used in this study are described in Table 1.

**Table 1:** Proteins used in this study. A, CPP- and CPPr-adaptors; B, cargo proteins. Sequences and additional information can be found in Supporting Information (S1 Fig).

	Name	Description (N-to-C)	Net Charge @ pH 7.0	Reference
A	TAT-CaM (2.0)	His-TAT-CaM	−19	[6]
	TAT-AUR-CaM	His-TAT-Aurein 1.2-CaM	−19	[7]
	TAT-LAH4-CaM	His-TAT-LAH4-CaM	−15	[7]
	TAT-NMR-CaM	His-TAT-naked mole rat CaM	−10	[10]
	GFP-CaM	His-GFP(+36)-CaM	+12	[7]
B	CGH9	His-CBS-GFP9-HiBiT	+17	This study
	CGH15	His-CBS-GFP15-HiBiT	+23	This study
	CGH20	His-CBS-GFP20-HiBiT	+28	This study
	CGH25	His-CBS-GFP25-HiBiT	+33	This study
	CGH36	His-CBS-GFP36-HiBiT	+44	This study

### Expression, purification and labeling

Proteins were expressed essentially as described [5,6] with minor modifications. Briefly, overnight cultures grown from single colonies were subcultured into 750 ml Luria-Bertani broth and grown with vigorous shaking at 37°C. At OD_600_ ~ 0.4, temperature was lowered to 30°C and cells were induced with 0.2 mM IPTG and growth continued for four hours. Cells were harvested by centrifugation at 10,000 x g and frozen at −80°C.

Purification of CPP-adaptors was performed as described [5]. Supercharged GFP cargos and GFP-CaM required protocol modification due to the tendency of the highly positive GFP cargos to precipitate in low salt. Cell pellets were thawed on ice, resuspended in 2M NaCl lysis buffer (50 mM Tris pH 8, 2 M NaCl, 2.5 mM imidazole, 10% glycerol) with Halt Protease Inhibitor Cocktail (ThermoFisher) added to 1x per manufacturer’s protocol. Cells were broken via passage through a French press at 20,000 psi and subjected to centrifugation at ~27,000 x g for 30 minutes at 4°C to pellet unbroken cells and debris. Clarified lysate was passed over a 1 or 5 ml Fast-flow HisTrap cobalt affinity column equilibrated with 1M NaCl Lysis Buffer (50 mM Tris pH 8, 1 M NaCl, 2.5 mM imidazole, 10% glycerol) using an FPLC system and washed with wash buffer (1 M NaCl lysis buffer, with 10 mM imidazole) until baseline absorbance was attained, after which protein was eluted with elution buffer (1 M NaCl lysis buffer with 250 mM imidazole). Cargo containing fractions were sampled for analysis then frozen in liquid nitrogen and stored at −80°C.

Although some GFP cargos were sufficiently pure for use after the HisTrap column, most cargos were further purified with Calmodulin Sepharose 4B matrix (Cytiva) by open phase affinity column purification. For this purification, IMAC elution fractions were quick thawed in water, placed on ice and brought to 1 mM CaCl_2_ and 2 M NaCl. These fractions were then loaded onto a 1–3 ml column equilibrated with 2 M NaCl lysis buffer with 1 mM CaCl_2_, washed with 5 column volumes of the same and eluted with 1 M NaCl lysis buffer containing 10 mM EDTA. GFP-containing fractions were sampled for purity analysis, frozen in liquid N_2_ and stored at −80°C. Fractions were later thawed, concentrated to a concentration above 100 µM on Amicon Ultra concentrators with a nominal 30 kDa molecular weight cutoff. In preparation for fluorescent labeling, concentrated cargos were desalted in 0.5 or 2 ml Zeba (Pierce) spin columns into 10 mM HEPES, pH 7.4, 1 M NaCl, 10% glycerol and 1 mM CaCl_2_. Desalted cargo protein was quantitated and treated with 0.4–0.6 mole of DyLight 650 NHS Esters (ThermoFisher) per mole of cargo. Dye removal columns (ThermoFisher) were then used to remove unreacted dye as recommended by the manufacturer. Protein concentration was estimated by Bradford and fluor incorporation estimated with the algorithm suggested by the manufacturer based on spectroscopic concentration analysis at 280 and 652 nm. This procedure produced incorporation efficiencies of about 0.2–0.3 dye molecules/cargo. Labeled cargos were aliquoted, frozen in liquid N_2_ and stored at −80°C until use. Core comparative experiments were all performed with a matched set of doubly purified cargos with incorporation of 0.17 to 0.23 fluors per cargo molecule.

### Cell Culture

Baby hamster kidney (BHK) cells were purchased from ATCC (#CCL-10). Cells were maintained in a 37°C, 5% CO_2_ environment in growth media consisting of DMEM, GlutaMax (containing + 4.5g/L D-glucose and 1.9 mM Ca^2+^ with no sodium pyruvate) and 5% fetal bovine serum (FBS). Cells were replated following trypsinization in chamber slides (Ibidi) in the same media 20–24 hours before use in cell penetration assays.

### Cell Penetration Assays

Internalization assays were modified from prior assays [5,6] to accommodate the high salt necessary to prevent cargo precipitation without producing large osmotic effects on cells. Complex assembly was performed at room temperature for the same reason.

Adaptors stored in a standard storage buffer (10 mM HEPES, pH 7.4, 150 mM NaCl, 10% glycerol, 1 mM CaCl₂) and GFP cargos (plus GFP-CaM) stored in high-salt buffer (10 mM HEPES, pH 7.4, 1 M NaCl, 10% glycerol, 1 mM CaCl₂) were thawed and diluted to appropriate concentrations in their respective storage buffers.

Complexes consisting of CGH cargos and adaptors were assembled at 100 × concentration by mixing 1 volume of cargo with 0.5 volumes of adaptor in their respective storage buffers, yielding a final salt concentration of 716 mM NaCl. Complexes involving CGH cargo and GFP-CaM were prepared in the 1 M NaCl storage buffer, followed by dilution with 0.5 volumes of the standard storage buffer yielding the same salt concentration.

Following assembly, complexes were incubated for 20–30 minutes at room temperature and then diluted using Glutamax with 5% FBS media, also at room temperature. Rapid dilutions were obtained by pipetting 400–1000 ul of media directly at the complex solution in the bottom of a microfuge tube followed by pipetting up and down several times. After 3–4 minutes at room temperature media containing complexes were microfuged at maximum velocity (>15,000 rcf) for 2 min to remove precipitates, briefly warmed in a 37°C bath (3–4 minutes) and transferred onto BHK cells from which growth media had just been removed. Cells were placed into an open-lid container in the incubator for 3 min and then, with lid closed, transferred to the confocal microscope. Care was taken to maintain conditions as close as possible to 37°C under 5% CO_2_ during transfer to a temperature and atmosphere-controlled chamber on the confocal microscope under the same conditions.

Confocal analysis was conducted using a Zeiss LSM 900 microscope at 400x magnification unless indicated. In all images, scale bars are 20 µm. Imaging parameters were optimized to maximize image quality, including the use of low laser intensity and Airyscan-optimized confocal settings. All imaging was conducted under standardized acquisition conditions using preconfigured channel settings from Zeiss for EGFP and AF647. EGFP was excited with a 488 nm laser, and emission was collected over the 410–617 nm range. DyLight 647 was excited with a 653 nm laser, with emission collected between 645–700 nm. Signals from both fluorophores were acquired within a single detection channel. GFP-labeled cargo exhibited strong binding to the slide surface, thus, images were captured at the mid-cell plane to minimize surface-related signal artifacts and avoid image degradation caused by precipitates. Typically, 4–6 wells with varying conditions, e.g., varied cargos and adaptors, were imaged in a repetitive cycle of 10–20 minutes. Imaging was begun as soon as practically possible (>8 min).

Time-course movies comparing cargo internalization in the presence and absence of TAT-CaM were conducted concurrently. Complexes prepared as above were carried to the confocal microscope with cells prepositioned in 8 well slides and added to wells immediately after removal of growth serum. Autofocus to maintain image clarity was set as fast as possible and imaging begun about 5 min after complex addition.

In general, confocal images were adjusted for presentation by raising signal intensity of the highest panel in an experiment to the top of the scale. Fluorescent signals were adjusted concordantly within each experiment although in some experiment conditions signal from GFP in each panel was adjusted to isolate yellow extracellular cargo from much redder intracellular cargo. Uniform background was also reduced in some panels. In all cases, representative images are shown from experiments repeated at least three times each producing multiple profiles from consecutive imaging cycles.

## Results

### Intrinsic internalization properties

As preparation for analysis of adaptor stimulated cargo internalization, it was necessary to demonstrate the intrinsic internalization properties of supercharged GFP cargos, initially designed as His-GFP-CBS-HiBiT fusion proteins. Although not used in the current study, the HiBiT sequence in association with a cytoplasmic fragment of luciferase produces light upon endosomal escape as part of a quantitative endosome escape assay. [22].

**Fig 1:**
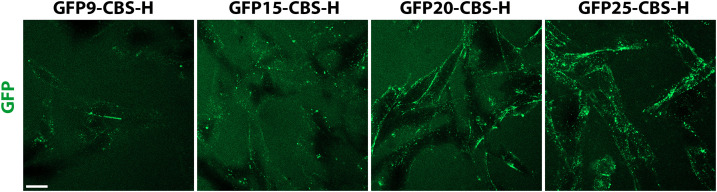
Intrinsic internalization of supercharged GFP cargos with His-GFP-CBS-HiBiT design. Cargos were diluted with media and added to cells grown at 37^°^C under 5% CO_2_ for 22-26 h on 8-well coverslip slides. Slides maintained under these conditions were transferred to the confocal microscope and imaged continuously for up to 90 min in a compartment that maintained cells at 37^°^C under 5% CO_2_. Internalization of His-GFP-CBS-HiBiT cargos with net GFP charge of +9, + 15, + 20 and +25. Representative profile (n = 3) showing GFP internalization 40-70 minutes after addition of media containing 200 nM cargo to cells. Images were taken on a Zeiss 900 microscope at 400x total magnification with collection of EGFP using preset windows provided by Zeiss (see Methods). Scale bars in all figures are 20 µm.

As expected, His-GFP-CBS-HiBiT cargos with variable positive charge have cell penetration abilities that increased as the positive charge of the GFP increased to net + 25 (Fig 1). As recently noted by the originating lab [16], supercharged GFP internalization does not appear to be robust. We recognized that these internalized GFPs never display perinuclear localization as observed for cargos that have attached fluorescent labels. We reasoned this might be due to loss of GFP fluorescence caused by lysosomal proteolytic cleavage, contrasting with slow loss of the incorporated label that would require very complete degradation. Unfortunately, the original His-GFP-CBS-HiBiT constructs proved too difficult to purify and label intact, necessitating a redesign to His-CBS-GFP-HiBiT (CGH) structure that is stable during purification and handling. To compare the behavior of variously charged CGH cargos, a matched set of identically purified cargos was labelled with limiting amine-reactive 650 DyLight NHS-Ester that incorporated only 0.2 dyes per CGH cargo molecule (S2 Fig).

**Fig 2:**
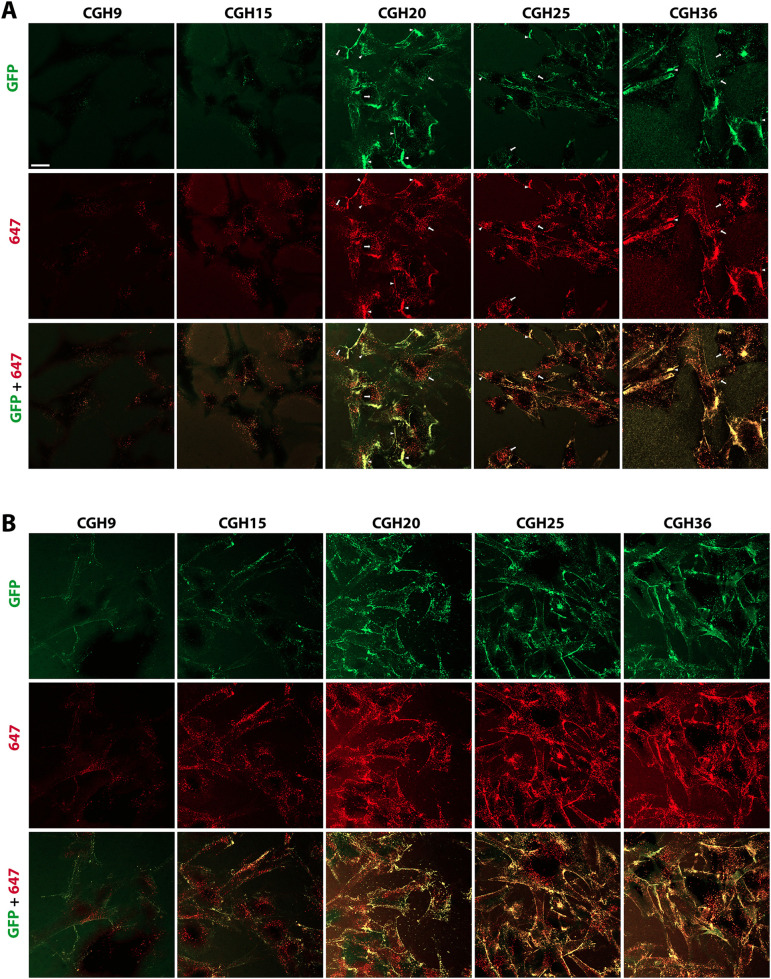
Intrinsic internalization of dual label supercharged GFP cargos with His-CBS-GFP-HiBiT (CGH) design. Cells treated with cargo containing media as in prior figure. **(A)** Representative profile (n = 5) showing internalization of 100 nM far-red (false red) labeled GFP cargos with the design His-CBS-GFP-HiBiT (CGH) and net GFP charge of +9, + 15, + 20, + 25 and +36 between 40-70 minutes. Arrows identify internal vesicles with red coloration due to acidic quenching of GFP signal. Arrow points identify CGH20 on the cell surface. **(B)** Representative profile (n = 5) showing internalization of far-red labeled CGH cargos at 400 nM cargo between 40-70 minutes. A matched set of CGH cargos labeled with DyLight 650-NHS Ester at ~0.2 moles dye per mole of cargo was used for direct comparison of 5 alternatively charged cargos. Images were taken on a Zeiss 900 microscope at 400x total magnification with collection of GFP and far-red fluorescence in a single track using preset windows provided by Zeiss (see Methods). Scale bars are 20 µm.

When the intrinsic internalization ability of these dual color CGH cargos was tested, the internalized vesicles displaying GFP fluorescence (Fig 2A, arrows in top panel) increased with cargo charge as had the original GFP constructs. However, the far-red label on the cargo showed much more intense intracellular localization (Fig 2A, middle panel). The composite image demonstrates the extent to which cargo in vesicles has lost most GFP fluorescence and appear red (to orange) due to far-red signal (Fig 2A, arrows). While pondering the extreme difference between GFP and false-red patterns, we realized that GFP fluorescence is pH-dependent and will be quenched below pH 6.2, as expected in most endosomal and lysosomal environments [23]. On the other hand, surface-localized complexes associated with the outer cell membrane are in the neutral pH extracellular environment where GFP retains full fluorescence and are thus yellow (Fig 2A, arrow points). The larger yellow patches found at cell edges represent disturbed membranes structures bound by cargo in contact with extracellular environment (Fig 2A, arrow points). Most small green punctates (Fig 2A, upper panel) colocalize with large red vesicles but some may be early endosomes that have yet to acidify. In any case, loss of most GFP fluorescence in acidic vesicular environments isolates false-red as vesicular localization, producing a readout that is extremely good evidence that cargo is intracellular and not on the cell surface. The dependence of internalization on cargo charge produces a consistent internalization profile. Focusing only on red vesicles in the dual color panel, it is visually apparent that CGH9 and CGH15 display less internalization than CGH25 and CGH36, a characteristic observed in all experiments at this concentration. Similarly, CGH20 displays intermediate internalization in almost all experiments, except for one case where it was similar to CGH15 and one where it was similar to CGH25. Initial plans included use of wild type GFP (net charge –5) as a CGH cargo, but its internalization was so far below scale that the panel was essentially dark, indicating negligible internalization and was therefore not investigated further.

The experiment above was performed at cargo concentrations of 100 nM (Fig 2A), which is 10-fold or more below the concentrations typically used with covalently linked CPP-cargos. When the same supercharged CGH cargos were used to treat cells at 400 nM (Fig 2B), the pattern of maximum internalization shifted so that moderately charged CGH cargos (CGH15 and CGH20) displayed a relative increase in internalization, with CGH20 reaching the same maximal level as the more charged cargos. For supercharged CGH cargos, intrinsic internalization correlates with cell surface association and both surface association and internalization appear saturable. This correlation suggests that charge increases internalization by increasing surface association.

### Internalization of CGH cargo series with a single adaptor

**Fig 3:**
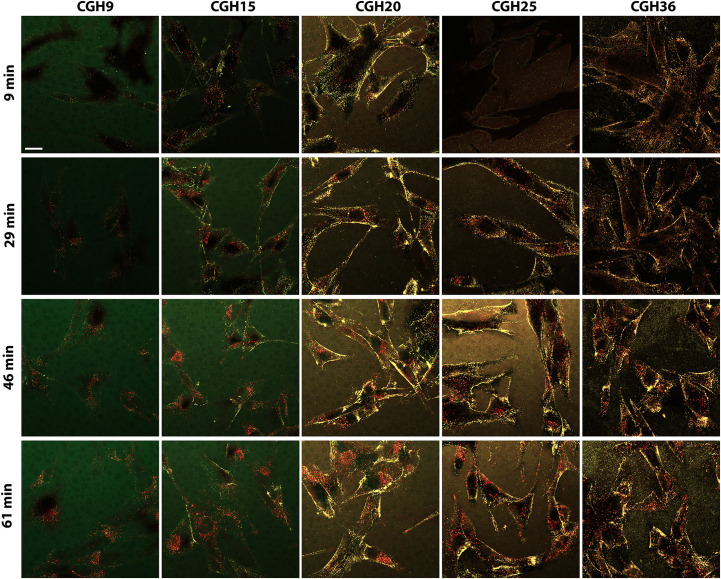
Time course of supercharged CGH cargo internalization in complex with TAT-CaM. Far-red labeled CGH cargos were combined with TAT-CaM as a premix (see Methods) so that dilution in media resulted in 100 nM cargo with 150 nM TAT-CaM. Adaptor/cargo complexes in media were handled as described in Methods, brought to 37°C and added to cells grown on coverslip slides at 37°C under 5% CO_2_ and then maintained under these conditions. To show the effect of time on cargo internalization, cells containing CGH25 complex were imaged first at 9 minutes after complex addition. Later sets were imaged beginning 29 min, 46 min and 61 min after complex addition with the cycle order of CGH25, CGH36, CGH9, CGH15, CGH20. For presentation in all images GFP is green and far-red 647 dye is false-red. Scale bar is 20 µm.

To examine the impact of adaptors on saturation effects and increased internalization of less positive cargos, relative internalization of 5 supercharged CGH cargos was compared in the presence of adaptors previously shown to be efficacious. Obviously, confocal images must be sequentially collected, which limits the number of wells that can be analyzed given the unavoidable time differential between the first and last images. Nevertheless, sequential sets create time series that initially show internalization increasing with longer cell treatment times. Such a time series for internalization of the CGH cargos at 100 nM in the presence of excess TAT-CaM is shown in Fig 3. In this experiment, the CGH25 containing well was imaged first, as soon as possible after complex addition (9 min) to show the low level of internalized CGH25 in the first image set relative to later sets. However, even in the set begun at 29 minutes, the image order is no longer obvious and sets begin at 46 and 61 minutes display very similar internalization profiles. Indeed, throughout these experiments, sets taken between 40–75 minutes are very similar, suggesting the internalization process has reached a stable profile showing the relative charge dependent internalization of the 5 cargo species in complex with TAT-CaM. Less positive, slowly internalizing cargos do display a relative increase at longer times, but this is a minor complication that does not alter relative cargo internalization within an experiment. To make the internalization data as simple and comparable as possible, we focus on the representative profiles taken between 40 and 75 minutes. The full time courses are included as supplemental figures to support the chosen pseudo-endpoint profile and because they often include additional information

**Fig 4:**
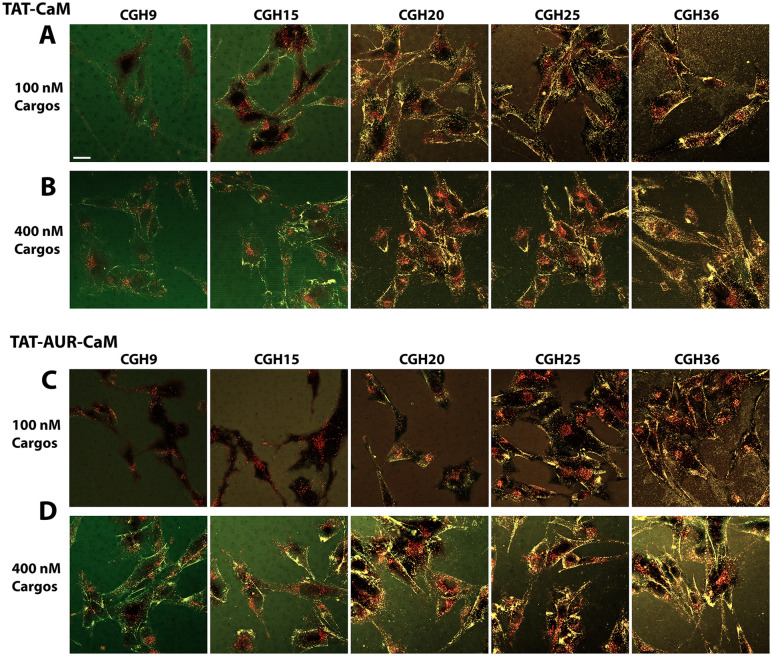
TAT-CaM and TAT-AUR-CaM do not drive maximal internalization of all CGH cargos. CGH cargos and adaptors were combined in a premix and then diluted in media followed by addition to cells and incubation at 37°C under 5% CO_2_. For these experiments TAT-CaM was in 1.5-fold excess of CGH cargos at **(A)** 100 nM and at **(B)** 400 nM, while TAT-AUR-CaM was in 1.1 fold excess of CGH cargos at **(C)** 100 nM and **(D)** 400 nM. Image sets presented are representative of profiles from 3 experiments with multiple profiles between 40-75 minutes. Scale bar is 20 µm.

As shown above, TAT-CaM has little ability to increase relative CGH9 internalization. Direct side-by-side comparison of TAT-CaM-mediated internalization of the 5 CGH cargos at 100 nM (Fig 4A and S3 Fig) and 400 nM (Fig 4B and S3 Fig) show profiles that match one another and are similar to the intrinsic internalization of the CGH series at 400 nM (Fig 1C). Thus 100 nM cargos complexed with TAT-CaM show increased internalization of moderately charged CGHs relative to intrinsic levels, while 400 nM CGH cargos with TAT-CaM show little change compared to cargos alone. The parallel experiment with the adaptor TAT-AUR-CaM showed even less adaptor effect as internalization profiles were similar to intrinsic internalization of the CGH series at both 100 nM (Fig 4C and S4 Fig) and at 400 nM (Fig 4D and S4 Fig). For both of these adaptors, limited ability to increase internalization of less charged cargos correlates with limited surface association.

**Fig 5:**
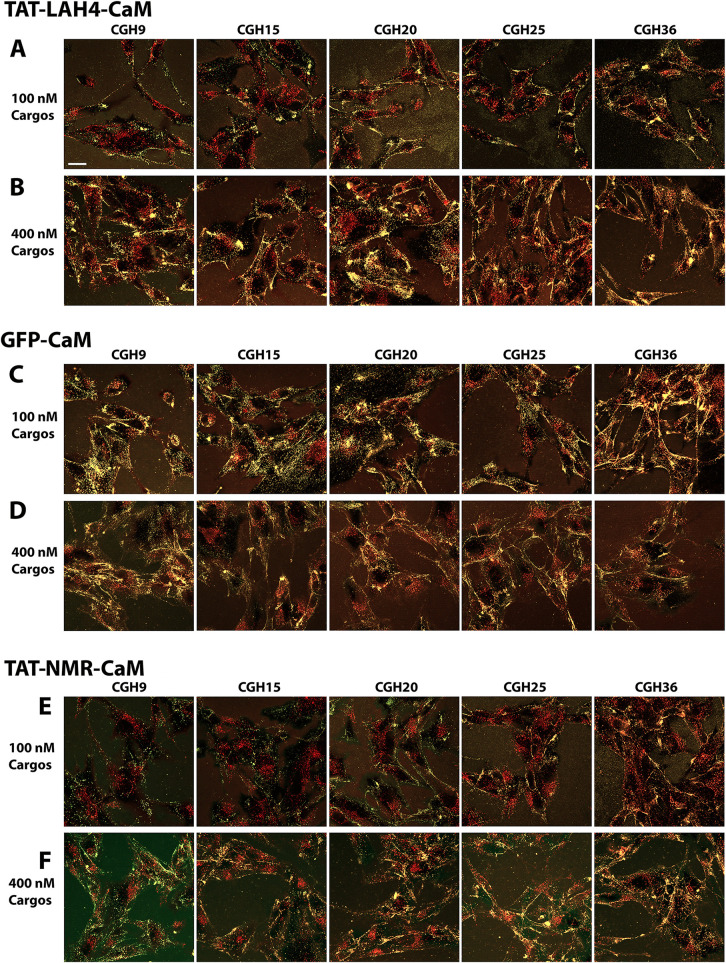
TAT-LAH4-CaM, TAT-NMR-CaM and GFP-CaM effect similar internalization regardless of cargo charge. CGH cargos and adaptors were combined in a premix and then diluted in media followed by addition to cells and incubation at 37°C under 5% CO_2_. For these experiments all adaptors were in excess of CGH cargos: 1.5x TAT-CaM with CGH cargos at **(A)** 100 nM and at **(B)** 400 nM; 1.1x for GFP-CaM with CGH cargos at **(C)** 100 nM and **(D)** 400 nM; and 1.1x for TAT-NMR-CaM with CGH cargos at **(E)** 100 nM and at **(F)** 400 nM. Image sets presented are representative of profiles from 3-4 experiments with multiple profiles between 40-75 minutes. Scale bar is 20 µm.

In contrast, for the adaptors TAT-LAH4-CaM, GFP-CaM and NMR-CaM, cargo internalization became much less sensitive to cargo charge. Most dramatically, TAT-LAH4-CaM internalized all CGH cargos at 100 nM (Fig 5A and S5 Fig) and at 400 nM (Fig 5B and S5 Fig), although the larger data set suggests CGH9 at 100 nM trended lower than other cargos. Robust internalization was accompanied by similar surface loading of all TAT-LAH4-CaM/CGH complexes.

Complexes of CGH cargos with GFP-CaM also showed increased surface association and charge independent internalization at both 100 nM (Fig 5C and S6 Fig). and 400 nM (Fig 5D and S6 Fig). Complicating interpretation, the increase in green signal due to the presence of two GFP domains within each complex made it more difficult to isolate red endosomal populations from dual color yellow populations. Decreasing green signal intensity failed to change potential intracellular punctates from yellow to red and it is likely that many are surface associated. Focusing on the red endosomes suggests a trend toward lower CGH9 internalization at 100 nM (Fig 5C).

In the parallel experiment with the TAT-NMR-CaM adaptor, less-charged cargos at 100 nM display lower internalization than more charged species (Fig 5E and S7 Fig), but internalization was charge independent for all cargos at 400 nM (Fig 5F and S7 Fig). For reasons that are unclear, variability in the internalization at 100 nM was high and the profile shown is a consensus choice. Nevertheless, at the higher concentration all three of these adaptors produced complexes where internalization can overcame less positive CGH cargo charge, resulting in similar levels of internalization.

The effects of individual adaptors on the panel of supercharged CGH cargos allowed relative comparison of each adaptor’s effects on a set of variably charged cargos. These data suggested a grouping of adaptors into those that poorly increase internalization of less supercharged cargos and those that appeared to stimulate internalization of these cargos. Importantly, the data does not demonstrate that the adaptors specifically increased internalization of any cargo.

### Individual CGH cargos internalized with an adaptor series

**Fig 6:**
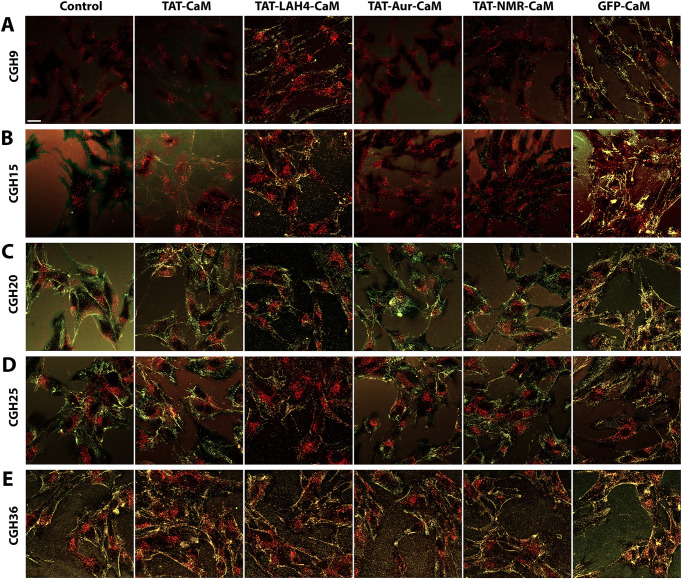
Comparison of intrinsic cargo internalization to that of cargo bound to TAT-CaM, TAT-LAH4-CaM, TAT-AUR-CaM, TAT-NMR-CaM and GFP-CaM. **(A)** CGH9 alone or combined with 5 adaptors in a premix was diluted in media to 100 nM with excess adaptor, followed by addition of the complex to cells and incubation at 37°C under 5% CO_2_ during imaging. Similarly, the cargos **(B)** CGH15, **(C)** CGH20, **(D)** CGH25 and **(E)** CGH36 either alone or combined with one of 5 adaptors in a premix was diluted in media to a concentration of 100 nM with excess adaptor. Image sets presented are representative of profiles from 3 experiments with multiple profiles between 40-75 minutes. Additional details provided with full experimental time course in the supplemental figures. Scale bar is 20 µm.

To demonstrate that an adaptor specifically increases internalization requires that the intrinsic internalization of a cargo be directly compared to adaptor induced internalization of that cargo. As adaptor induced internalization will generally be hidden if the CGH cargos are already at saturating concentration, specific internalization was tested at 100 nM cargo where most CGH cargos are below intrinsic saturation levels. To make the effectiveness of the adaptors comparable, the intrinsic internalization of one CGH cargo was simultaneously compared to internalization of that cargo in complex with all 5 adaptors in moderate excess. For each cargo the most representative profiles from multiple experiments between 40–70 minutes were selected and combined into a single composite image (Fig 6A-E).

For each of these experiments, the first column shows the intrinsic internalization of each cargo alone compared to the simultaneously assayed complexes of adaptors with that cargo. The most striking feature of this image is the robust specific internalization of most CGH cargos by TAT-LAH4-CaM (Fig 6, column 3) including dramatic increases for CGH9 (Fig 6A and S8 Fig) and CGH 15 (Fig 6B and S9 Fig) relative to intrinsic levels seen in column 1. Internalization of CGH20 (Fig 6C and S10 Fig) and CGH25 (Fig 6D and S11 Fig) also appeared to display increases above intrinsic levels, although modest increases near maximal levels have low confidence.

By comparison, TAT-CaM was less effective with the less charged cargos and may have decreased internalization of CGH9 (Fig 6A, col 2) below intrinsic levels. On the other hand, TAT-CaM specifically increased internalization of more positively charged cargos; however, the effects were modest. The parallel experiment with the adaptor TAT-AUR-CaM did not show consistently increased internalization with any cargo (Fig 6, col 3). The relative ineffectiveness of TAT-CaM and TAT-Aur-CaM with less charged cargos is consistent with their limited ability to alter the cargo charge response profiles (Fig 4).

Although GFP-CaM appeared to stimulate internalization of less-positive CGH cargos in the profiles above (Fig 5C and 5D), the data here indicate the situation is more complicated. While GFP-CaM (Fig 6, col 6) does increase CGH9 (Fig 6) and CGH15 (Fig 6B) internalization, the adaptor decreased internalization of the highly charged CGH25 (Fig 6D) and CGH36 (Fig 6E and S12 Fig) below intrinsic levels. As a consequence of these divergent effects observed for CGH cargos, GFP-CaM produces similar internalization of all CGH cargos as observed above (Fig 5C). The effect of TAT-NMR-CaM on internalization of the CGH cargos was similar to GFP-CaM, in that TAT-NMR-CaM decreased internalization of the most positive cargos, CGH25 and CGH36 (Fig 6) and produces a less charge dependent profile as reported above (Fig 5E). While inhibitory effects of TAT-NMR-CaM probably play a role in the charge independent internalization profile observed above at higher complex concentrations (Fig 5F), this issue was not pursued as poor functionality and inconsistent behavior (Fig 5E discussion) suggested little benefit.

Because TAT-LAH4-CaM specifically increased internalization of less charged cargos (Fig 6), this adaptor produced comparable internalization of all cargos regardless of charge even at 100 nM (Fig 5A). Consequently, the experiments presented in Fig 6 share a common scale, as intensity of TAT-LAH5-CaM internalized cargos have been set to similar levels (Fig 6 col. 3). Even though the columns in this figure are composed of data from 5 separate experiments, they reproduce the charge response profiles of the cargos alone and in complex with each adaptor (Figs. 1–5 100 nM profiles). Gross conservation is readily apparent; however, more subtle differences required additional verification.

**Fig 7:**
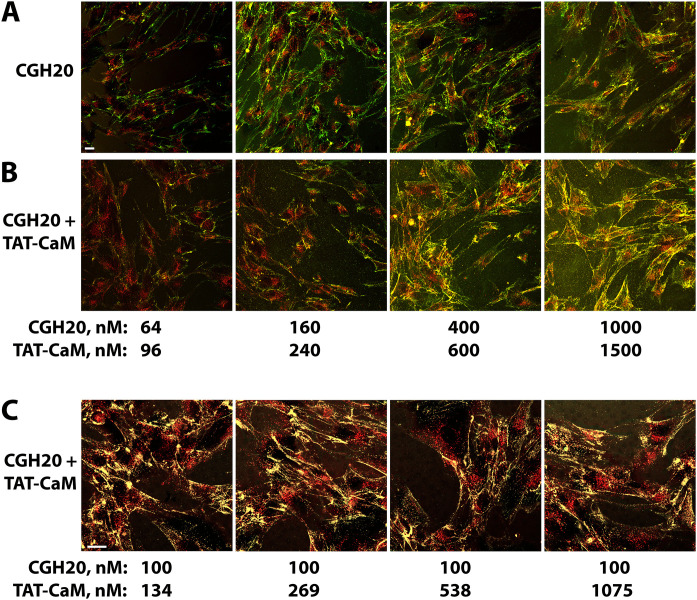
TAT-CaM increases internalization of CGH20 across a range of complex concentrations. CGH20 and TAT-CaM were combined in a premix and then diluted in media to the concentrations indicated, followed by addition to cells and incubation at 37°C under 5% CO_2_. Comparison of CGH20 internalization **(A)** without or **(B)** with TAT-CaM at a 1.5-fold excess of CGH20 cargos. Images ± TAT-CaM taken consecutively at each concentration. Panels images at 200x **(C)** Internalization of 100 nM CGH20 with 4 concentrations of excess TAT-CaM. Image sets presented are representative of profiles from 3 experiments with profiles between 40-75 minutes. Imaging order: high to low concentration. Scale bars are 20 µm.

While TAT-CaM often requires µM adaptor/cargo concentrations for specific cargo internalization [7], the data above suggest the adaptor can be effective at lower concentrations with positive cargos. To confirm the ability of TAT-CaM to increase internalization of positive cargos, we directly compared internalization of CGH20 without (Fig 7A) and with TAT-CaM (Fig 7B) across a range of concentrations. Indeed, TAT-CaM moderately increased CGH20 internalization at all concentrations including 400 and 1000 nM, strongly suggesting TAT-CaM produces internalization above maximal intrinsic internalization of this cargo. The analysis was performed at lower magnification (200x) to increase confidence in the comparison even though isolation of intracellular red signal is more difficult. We also demonstrated that TAT-CaM in excess of CGH20 produced similar internalization across a range of adaptor concentrations (Fig 7C).

**Fig 8:**
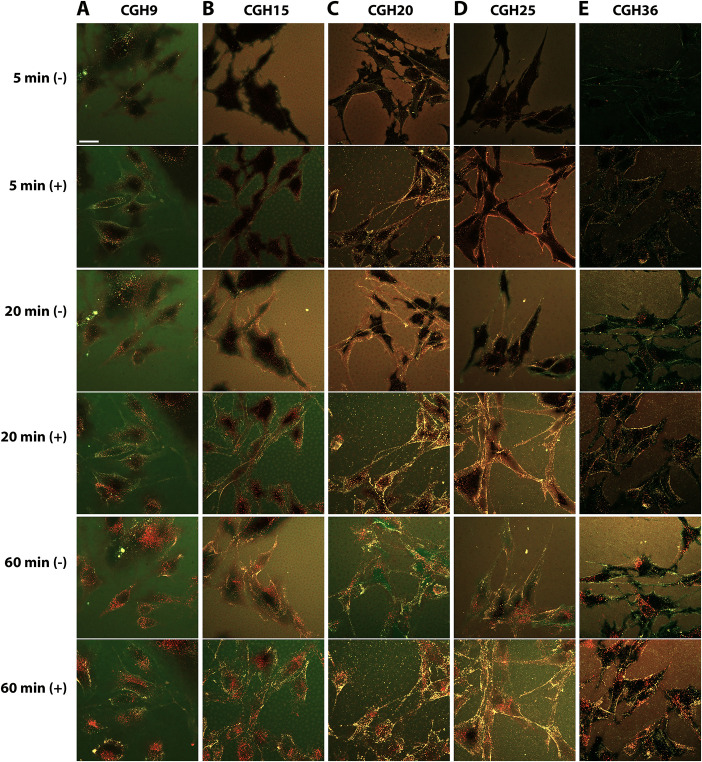
Selected still images from simultaneous movies of each cargo with or without TAT-CaM. For each cargo, movies showing internalization were initiated by addition of media with 400 nM CGH cargo without (-) or with (+) 600 nM TAT-CaM. These movies were obtained by alternative imaging of (-) and (+) wells for at least 60 minutes. Paired still images at selected times during the movies show internalization of **(A)** CGH9, **(B)** CGH15, **(C)** CGH20, **(D)** CGH25 and **(E)** CGH36, without or with TAT-CaM. Full movies are available as respective supplemental figure (S18-22 Fig). Scale bar is 20 µm.

The experiments above are based on endpoints beyond 40 minutes of incubation. However, this approach sacrifices information on the rate of cargo entry and has limited ability to show differences when internalization approaches maximal levels at the pseudo-endpoint. While it is generally impractical to analyze internalization using time courses, limited validation of the endpoint assay was obtained for TAT-CaM complexed with the 5 CGH cargos by simultaneously imaging internalization of a single cargo with and without TAT-CaM. We chose the 400 nM cargo concentration to clearly demonstrate TAT-CaM can stimulate internalization beyond intrinsic levels. As shown in the movies (S13–17 Figs) and in static images of selected time points (Fig 8A-E), cargos other than CGH9 gain entry to intracellular vesicles more rapidly in the presence of TAT-CaM. TAT-CaM stimulated entry of CGH20 and CGH25 was particularly fast and corresponded with a clear increase in cargo internalization at 60 minutes. While less dramatic early internalization of CGH15 and CGH36 was also increased by TAT-CaM and endpoint levels at 60 min suggested modest specific internalization. In contrast, the early internalization of CGH9 was not significantly increased by TAT-CaM and trended below that of cargo alone at the 60 min endpoint. Multiple lines of evidence demonstrate that TAT-CaM failed to stimulate internalization of CGH9 while increasing the rate and extent of internalization for more positive cargos.

**Fig 9:**
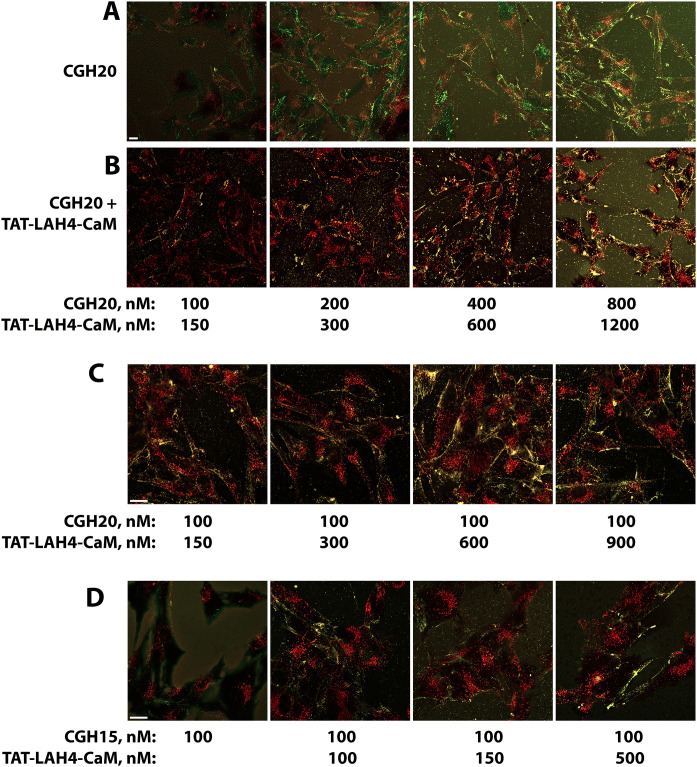
TAT-LAH4-CaM specifically increases internalization of CGH20 across a range of complex concentrations. CGH20 and TAT-LAH-CaM were combined in a premix and then diluted in media to the concentrations indicated, followed by addition to cells and imaging at 200x magnification. Comparison of CGH20 internalization at 100, 200, 400 and 800 nM **(A)** without or **(B)** with a 1.5-fold molar excess TAT-LAH4-CaM. Images ± TAT-CaM taken consecutively at each concentration. **(C)** Internalization of 100 nM CGH20 with excess TAT-LAH4-CaM at 150, 300, 600 and 900 nM. **(D)** Internalization of 100 nM CGH15 without or with TAT-LAH4-CaM at 100, 150 and 500 nM. Image sets presented are representative of profiles from 3 experiments with profiles between 40-75 minutes. Imaging order: high to low concentration. Scale bars are 20 µm.

In contrast to TAT-CaM, TAT-LAH4-CaM was effective at stimulating internalization of CGH cargos regardless of GFP charge state. Given the ability of TAT-CaM to stimulate CGH20 internalization beyond maximal intrinsic levels, we wished to confirm that TAT-LAH4-CaM had this same ability. Dose response experiments comparing CGH20 (Fig 9A) alone to TAT-LAH4-CaM/CGH20 complexes (Fig 9B) showed increased CGH20 internalization at all complex concentrations. TAT-LAH4-CaM stimulated internalization at 100 nM as shown above (Fig 6C), but did so to a greater extent at 400 nM where CGH20 displayed maximal intrinsic internalization (Fig 2A). We also demonstrate that excess TAT-LAH4-CaM across a range of concentration produced similar internalization of complexes with 100 nM CGH20 (Fig 9C) or with 100 nM CGH15 (Fig 9D).

**Fig 10:**
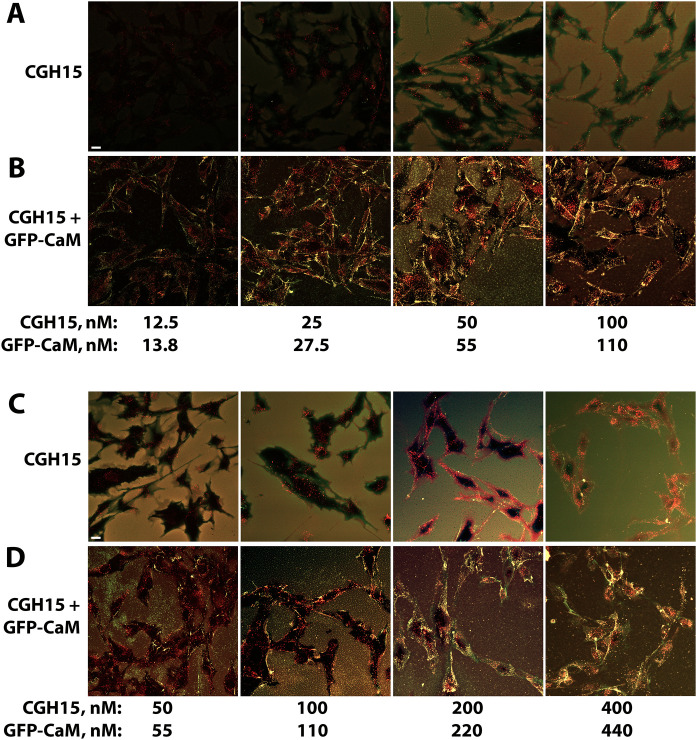
Dose dependence of GFP-CaM/CGH15 internalization at low and high cargo concentrations. GFP-CaM and CGH15 were combined in a premix and then diluted in media to the concentrations indicated, followed by addition to cells and incubation at 37ºC under 5% CO2. Low dose comparison of GFP-CaM/CGH15 internalization at 12.5, 25, 50 and 100 nM without **(A)** or with **(B)** a 1.1-fold excess of GFP-CaM. High dose comparison of CGH internalization at 50, 100, 200 and 400 nM without **(C)** or with **(D)** a 1.1-fold excess of GFP-CaM. Imaging order: high to low concentration. Images ± GFP-CaM taken consecutively at each concentration with 200x magnification. Scale bars are 20 µm.

Adaptors TAT-NMR-CaM and GFP-CaM were designed to mitigate the negative charge of CaM and are very effective with some cargos [7,10] despite the inhibitory characteristics shown in Fig 6. We suspected the GFP-CaM/CGH complexes were saturating the cell surface to the point of inhibiting internalization and wanted to determine if reduced complex concentration would increase CGH internalization. The CGH15 cargo was used to avoid saturating intrinsic internalization and because GFP-CaM stimulated internalization of CGH15 at 100 nM (Fig 6, col 6). As expected, across a range of low concentrations (12.5 nM-100 nM) where CGH15 alone internalizes poorly (Fig 10A), association with GFP-CaM produced strong specific increases in CGH15 internalization (compare Fig 10A to 10B). However, at higher concentrations, GFP-CaM/CGH15 internalization increased very little (Fig 10D), while intrinsic CGH15 internalization increased dramatically (Fig 10C). Thus, despite GFP-CaM induced CGH15 internalization at low concentration, the adaptor prevented even intrinsic levels of CGH15 internalization at higher concentrations. The similarity of GFP-CaM/CGH15 internalization from 25 nM to 400 nM suggests high surface affinity and surface saturation limit internalization, rather than high dose inhibition. The effect of 1.5 or 2 fold excess GFP-CaM above CGH15 concentration was also tested and did not appear to increase internalization (Morris, unpublished). Without any clear benefit, excess GFP-CaM increased yellow surface labelling and yellow punctates greatly complicating separation of intracellular and surface complexes.

## Discussion

The CPP-adaptor system was developed to solve the problem of endosomal entrapment by decoupling cargos from CPPs that remain bound to endosomal membranes and trafficked to lysosomes. However, upon application of the adaptor system, it became clear that intracellular separation of cargo from adaptor had numerous other advantages. Establishing the ability of an adaptor to specifically increase internalization is a core part of our internalization assay and this feature soon revealed that cargos with highly positive net charges displayed intrinsic internalization. A particularly dramatic example was provided by the very positive cargo Cas9, the protein component of CRISPR. Labeled TAT-CaM and labeled CBS-Cas9 internalized readily at concentrations more than 10-fold below TAT-CaM alone [7]. In other words, cargo mediated adaptor internalization.

Obviously, this challenged interpretations of many CPP internalization studies as the role of cargo is rarely tested. Our data showing that increasingly positive cargo charge increased internalization were strangely consistent, given that context specific effects must occur. This pointed to the need for systematic investigation of the effect of positive cargo charge and the interaction of cargo charge with CPP- and CPP-adaptor-mediated internalization.

### Experimental design

To address the effect of cargo charge on internalization, we designed cargos based on a set of variant GFPs which had already been shown to display increasing internalization with increasing positive charge [16]. As reported, we found increasing GFP charge from +9 to +25 increased internalization, though internalization appeared to be poor. When more stable redesigned CGH cargos were labeled with a covalently attached fluor, the label on these cargos revealed strong internalization despite a near absence of intracellular GFP fluorescence. The failure of intracellular GFP to fluoresce is mostly due to the acidic environment of intracellular endosome, which is generally below pH 6.2, where GFP fluoresces poorly.

While increased signal was important, the ability to distinguish dually fluorescing extracellular cargo from intracellular cargo that lacked strong GFP fluorescence was even more valuable. Confocal imaging could then distinguish yellow cargo present on the slide and outside the cell surface from red cargo inside the cell, removing the need for washing and the associated multilayered artifacts.

While the dual fluor CGH cargos provided a powerful system for investigation of CPP mediated internalization, there are complications that seem to be intrinsic to the supercharged GFPs. Most significantly, cargos with highly charged GFPs tend to precipitate. Limiting precipitation required high concentrations of NaCl from the beginning of purification through dilution of adaptor/cargo complexes in media. High concentrations of cargos were then required so that large dilutions could limit salt addition to cell media. Even so, some cargos and some complexes produced problematic levels of precipitation during imaging. Temperature also impacted internalization and experiments here were performed with minimal cooling of cells during handling and treatment. Given the difficulty of individual experiments and the complexity of the experimental space, systematic approaches were limited to variables that seemed particularly important. It is fortuitous that excess adaptor had little effect on complex internalization across a range of concentrations, removing one problematic variable.

### Intrinsic internalization

Because confocal microscopes cannot simultaneously analyze multiple wells, a pseudo end point between 40 and 70 minutes was used as the internalization profile was surprisingly constant during this period. Initial work with the CGH cargos revealed charge and concentration dependent, saturable, surface association that usually correlated with maximal internalization. *A priori*, this suggests a limit on surface association due to positive charge and thus a limit on charge dependent internalization. The relatively stable internalization profile observed across the CGH states between 40 and 70 minutes may involve exhaustion of a dominant internalization process. However, the complex mechanisms that produce relatively stable profiles are beyond the scope of the current study.

### Adaptor effects

Given the dominant role of net cargo charge on intrinsic CGH internalization, the diversity of responses cargo charge produced by a limited set of 5 adaptors shows that cargo charge is not the only determinant controlling internalization. In particular, TAT-CaM specifically increased internalization of most CGH cargos without consistently increasing cargo localization at the cell surface. In addition, TAT-CaM was ineffective at increasing either surface localization or internalization of CGH9, while increases in CGH15 internalization occurred with little surface localization. Endpoint assays showed that TAT-CaM was effective with CGH20, resulting in strong internalization that appears to reach a common maximal level for the more positive cargos. Specific stimulation of CGH25 and CGH36 internalization was difficult to assess using the endpoint assay, but movies of internalization with and without adaptor showed that TAT-CaM specifically increased the rate of internalization of CGH15, CGH20, CGH25 and CGH36.

The ability of TAT-CaM to increase the rate of internalization of more moderately positive cargos such as CGH20 and CGH25 was striking. Further, the stimulatory effect of TAT-CaM on CGH20 internalization occurred across a range of complex concentrations including some far below the µM range typically used with this adaptor. In contrast, TAT-CaM was very ineffective with the cargo CGH9, a cargo that displayed very little intrinsic internalization or membrane association. Consistent with the inability of TAT-CaM to increase surface association, a parsimonious explanation for this charge-dependent difference is that TAT-CaM stimulates internalization of cargos associating with the plasma membrane but has limited ability to recruit cargos to the cell surface.

In dramatic contrast, TAT-LAH4-CaM was impressive in driving maximal internalization of all cargos regardless of charge even at very low complex concentration. This ability correlates with a strong phenotype involving recruitment of CGH cargos of all charge states to the plasma membrane. While the LAH4 sequence was originally identified as an EP, later evidence revealed CPP activity [19,24]. The TAT-LAH4-CaM adaptor was identified while investigating EP functionality within the context of inclusion in both the adaptor (TAT-EP-CaM) and a maltose binding protein cargo (CBS-EP-MBP) [7]. As TAT-LAH4-CaM was able to internalize negatively charged MBP cargos it is not too surprising that TAT-LAH4-CaM increased surface association and internalization of neutral CGH15 and CGH9. Further, the TAT sequence in TAT-LAH4-CaM may be activating internalization as the adaptor stimulated CGH20 internalization even at high concentrations. TAT-AUR-CaM was developed in the same study and included here because the Aurein 1.2 sequence is endolytic [25] and because a CBS-AUR-MBP cargo produced a strong negative effect on GFP-CaM internalization associated with peripheral endosomal trapping [7]. While TAT-AUR-CaM may have had some inhibitory effects on CGH cargo internalization, the behavior was too modest to justify further study.

Both TAT-NMR-CaM and GFP-CaM represented successful efforts to improve adaptor function by increasing the net positive charge of adaptors [7,10]. The dominance of charge and concentration in producing maximal intrinsic internalization of CGH cargos, correlates with surface loading of a cargo. Consistent with this idea, GFP-CaM localized all of the CGH cargos to the cell surface and internalized these cargos to about the same extent at both 100 and 400 nM. Unfortunately, this equalization had as much to do with inhibition of internalization for more charged CGH cargos as it had to do with modestly increased internalization observed with CGH9 and CGH15. We have observed inhibition by high concentrations of adaptor/cargo complexes in the past, including one instance involving GFP-CaM [7]. However, the concentration dependence of GFP-CaM/CGH15 on complex internalization revealed strikingly little dose effect from 25 to 400 nM, inconsistent with high dose inhibition. On the other hand, dose independent internalization is consistent with strong surface association establishing maximal internalization even at very low concentrations. Unfortunately, maximal GFP-CaM-induced internalization is well below maximal intrinsic internalization of CGH15, revealing that some characteristic of the GFP-CaM/CGH15 complex is problematic for internalization. While it would not seem to be necessary, robust internalization at low concentration may require a trigger that stimulates the internalization process. Active investigation continues in this direction.

## Conclusion

Application of CPP-adaptor technology to a set of differentially charged cargos has produced a diverse set of outcomes that suggest the power this approach may deliver. At its core, the power of CPP-adaptors lies in the flexibility inherent to separation of the CPP function from the cargo, which allows different functionalities to be placed within the CPP and the cargo. We have uncovered an apparent limit to the maximal internalization that can be achieved by positive charge alone and shown that adaptors including TAT-CaM and TAT-LAH4-CaM may increase internalization to a higher level. We have also shown adaptors with high affinity for the cell surface can dramatically reduce the concentrations at which cargos will internalize. The cargos used in this study were designed with the HiBiT sequence to assay endosomal escape. Those studies were delayed by the need to maximize CPP-adaptor internalization so that endosomal escape assays so that signals are high enough to be readily assayable. In our recent studies, we have barely scratched the surface possible with CPP-adaptors using limited sets of cargos and adaptors. The next endeavor will be to measure and optimize parameters that enhance endosomal escape.

## Supporting information

S1 Fig Sequences and characteristics of GFP Cargos. Supercharged GFP sequences were taken from Thompson et al (2012) (Reference #16).
(PDF)

S2 Fig Matched CGH cargo purification, labeling and gel analysis. As described in Methods, CGH cargos expressed in E coli were extracted, purified on 5 ml cobalt columns and then again using open phase CaM column matrix. Eluted protein was concentrated, desalted into buffer with 10% glycerol and 1M NaCl and labeled with 0.4 mol Dylight NHS ester per mol of protein. Following dye removal, the cargos were quantitated by Bradford and 2 µg of each was run on a 4–20% precast biorad gel, which was stained by Coomassie and imaged. An unaltered, uncropped photograph of the gel used to make this image is available at https://doi.org/10.6084/m9.figshare.32736102.
(PDF)

S3 Fig Time series comparing internalization of CGH cargos in complex with TAT-CaM. Far-red labeled CGH cargos (CGH9, CGH15, CGH20, CGH25, CGH36) were combined with TAT-CaM, diluted in media and used to treat cells as described in the Methods. One well included only CGH20 as a limited specificity control that is strictly comparable only to internalization of the CGH20/TAT-CaM complex. **(A)** Time series showing internalization of 100 nM CGH cargos with 150 nM TAT-CaM (n = 3), alongside cells treated with 100 nM CGH20 alone (far right) as a limited control that shows intrinsic internalization at this concentration. Profiles of cargo internalization beginning 48 min, 69 min and 101 min after complex addition (48 min used in Fig 4A). Imaging order was not consistent between profiles. **(B)** Time series showing internalization of 400 nM CGH cargos with 600 nM TAT-CaM (n = 4) alongside cells treated with 400 nM CGH20 alone as a limited control that shows intrinsic internalization at this concentration (see Fig 1C). Profiles of cargo internalization beginning 11, 26, 46 and 65 min after complex addition (46 min used in Fig 4B). Imaging order: CGH36, CGH25, CGH9, CGH15, CGH20 and CGH20 alone.
(PDF)

S4 Fig Time series comparing internalization of CGH cargos in complex with TAT-AUR-CaM. Far-red labeled CGH cargos combined with TAT-AUR-CaM were diluted in media then added to cells as in Methods. **(A)** Time series showing internalization of 100 nM of CGH cargos with 150 nM TAT-AUR-CaM, alongside cells treated with 100 nM CGH20 alone as a limited specificity control (n = 3). Profiles of cargo internalization beginning 16, 30, 47 and 62 min after complex addition (47 min used in Fig 4C). Imaging order: CGH9, CGH15, CGH20, CGH20 alone, CGH36 and CGH25. **(B)** Time series showing internalization of 400 nM CGH cargos with 600 nM TAT-AUR-CaM, alongside cells treated with 400 nM CGH20 alone as a limited specificity control (n = 3). Profiles of cargo internalization beginning 10, 27, 42 and 59 min after complex addition (42 min used in Fig 4D). Imaging order: CGH36, CGH25, CGH9, CGH15, CGH20 and CGH20 alone.
(PDF)

S5 Fig Time series comparing internalization of CGH cargos in complex with TAT-LAH4-CaM. Cells were treated with complexes of the CGH cargos and TAT-LAH4-CaM, along with a limited specificity control containing CGH20 alone. **(A)** Time series showing internalization of 100 nM of CGH cargo with 150 nM TAT-LAH4-CaM, alongside a control with 100 nM CGH20 alone (n = 3). Profiles of cargo internalization beginning 13, 33, 46 and 60 min after complex addition (46 min used in Fig 5A). Imaging order: CGH9, CGH15, CGH20, CGH20 alone, CGH36 and CGH25. **(B)** Time series showing internalization of 400 nM CGH cargos with 600 nM TAT-LAH4-CaM, alongside a control with 400 nM CGH20 alone (n = 3). Profiles of cargo internalization beginning 13, 29, 44 and 61 min after complex addition (44 min used in Fig 5B). Imaging order: CGH9, CGH15, CGH36, CGH25, CGH20 and CGH20 alone.
(PDF)

S6 Fig Time series comparing internalization of CGH cargos in complex with GFP-CaM. Cells were treated with complexes of the CGH cargos and GFP-CaM, along with a limited specificity control containing CGH20 alone. **(A)** Time series showing internalization of 100 nM of CGH cargo with 110 nM GFP-CaM, alongside a control with 100 nM CGH20 alone (n = 3). Profiles of cargo internalization beginning 10, 33, 46 and 68 min after complex addition (46 min used in Fig 5C). **(B)** Time series showing internalization of 400 nM CGH cargos with 440 nM GFP-CaM, alongside a control with 400 nM CGH20 alone (n = 4). Profiles of cargo internalization beginning 11, 27, 44 and 64 min after complex addition (44 min used in Fig 5D). Imaging order: CGH9, CGH15, CGH20, CGH20 alone, CGH36 and CGH25.
(PDF)

S7 Fig Time series comparing internalization of CGH cargos in complex with TAT-NMR-CaM. Cells were treated with complexes of the CGH cargos and TAT-NMR-CaM, along with a limited specificity control containing CGH20 alone. **(A)** Cells treated with 100 nM of CGH cargo with 110 nM TAT-NMR-CaM, alongside a control with 100 nM CGH20 alone (n = 5). Profiles of cargo internalization beginning 12, 30, 46 and 59 min after complex addition (46 min used in Fig 5E). **(B)** Cells treated with 400 nM CGH cargos plus 440 nM TAT-NMR-CaM alongside a control with 400 nM CGH20 alone (n = 4). Profiles of cargo internalization beginning 10, 27, 44 and 66 min after complex addition (44 min used in Fig 5F). Imaging order: CGH9, CGH15, CGH20, CGH20 alone, CGH36 and CGH25.
(PDF)

S8 Fig Time series directly comparing internalization of CGH9 alone and in complex with adaptors. Internalization of 100 nM CGH9 alone or with 250 nM TAT-CaM, TAT-LAH4-CaM and TAT-AUR-CaM or 110nM TAT-NMR-CaM and GFP-CaM. Representative experiment showing profiles of cargo internalization imaged beginning 20, 35 and 47 min after complex addition (47 min used in Fig 6A). (n = 3).
(PDF)

S9 Fig Time series directly comparing internalization of CGH15 alone and in complex with adaptors. Internalization of 100 nM CGH15 alone or with 150 nM TAT-CaM, TAT-LAH4-CaM, TAT-AUR-CaM, TAT-NMR-CaM and GFP-CaM. Representative experiment showing profiles of cargo internalization imaged beginning 15, 30 and 50 min after complex addition (50 min used in Fig 6B). (n = 3).
(PDF)

S10 Fig Time series directly comparing internalization of CGH20 alone and in complex with adaptors. Internalization of 100 nM CGH20 alone or with 150 nM TAT-CaM, TAT-LAH4-CaM, TAT-AUR-CaM, TAT-NMR-CaM and GFP-CaM. Representative experiment showing profiles of cargo internalization imaged beginning 24, 39 and 52 min after complex addition (52 min used in Fig 6C). (n = 4).
(PDF)

S11 Fig Time series directly comparing internalization of CGH25 alone and in complex with adaptors. Internalization of 100 nM CGH25 alone or with 500 nM TAT-CaM, TAT-LAH4-CaM and TAT-AUR-CaM or 110nM TAT-NMR-CaM and GFP-CaM. Representative experiment showing profiles of cargo internalization imaged beginning 13, 38 and 58 min after complex addition (58 min used in Fig 6D). (n = 4).
(PDF)

S12 Fig Time series directly comparing internalization of CGH36 alone and in complex with adaptors. Internalization of 100 nM CGH36 alone or with 500 nM TAT-CaM, TAT-LAH4-CaM and TAT-AUR-CaM or 110nM TAT-NMR-CaM and GFP-CaM. Representative experiment showing profiles of cargo internalization imaged beginning 40 and 56 min after complex addition (56 min used in Fig 6E). (n = 3).
(PDF)

S13 Fig Simultaneous video comparison of the internalization of 400 nM CGH9 ± 600 nM TAT-CaM. 650-CGH9/TAT-CaM complexes were set up and diluted in media as in other experiments. Complexes in media at 37ºC under 5% CO_2_ were brought to the confocal microscope and added to neighboring slide wells immediately following removal of growth media. Imaging was begun immediately after the autofocus was set at about 5 minutes and alternate imaging of paired wells was continued for at least 60 min. Movies are set for simultaneous viewing of each 650-CGH9 ± TAT-CaM for 60 min. Initiate by starting slide presentation.
(PPTX)

S14 Fig Simultaneous video comparison of the internalization of 400 nM CGH15 ± 600 nM TAT-CaM. 650-CGH15/TAT-CaM complexes were set up and diluted in media as in other experiments. Complexes in media at 37ºC under 5% CO_2_ were brought to the confocal microscope and added to neighboring slide wells immediately following removal of growth media. Imaging was begun immediately after the autofocus was set at about 5 minutes and alternate imaging of paired wells was continued for at least 60 min. Movies are set for simultaneous viewing of each 650-CGH15 ± TAT-CaM for 60 min. Initiate by starting slide presentation.
(PPTX)

S15 Fig Simultaneous video comparison of the internalization of 400 nM CGH20 ± 600 nM TAT-CaM. 650-CGH20/TAT-CaM complex were set up and diluted in media as in other experiments. Complexes in media at 37ºC under 5% CO_2_ were brought to the confocal microscope and added to neighboring slide wells immediately following removal of growth media. Imaging was begun immediately after the autofocus was set at about 5 minutes and alternate imaging of paired wells was continued for at least 60 min. Movies are set for simultaneous viewing of each 650-CGH20 ± TAT-CaM for 60 min. Initiate by starting slide presentation.
(PPTX)

S16 Fig Simultaneous video comparison of the internalization of 400 nM CGH25 ± 600 nM TAT-CaM. 650-CGH25/TAT-CaM complexes were set up and diluted in media as in other experiments. Complexes in media at 37ºC under 5% CO_2_ were brought to the confocal microscope and added to neighboring slide wells immediately following removal of growth media. Imaging was begun immediately after the autofocus was set at about 5 minutes and alternate imaging of paired wells was continued for at least 60 min. Movies are set for simultaneous viewing of each 650-CGH25 ± TAT-CaM for 60 min. Initiate by starting slide presentation.
(PPTX)

S17 Fig Simultaneous video comparison of the internalization of 400 nM CGH36 ± 600 nM TAT-CaM. 650-CGH36/TAT-CaM complexes were set up and diluted in media as in other experiments. Complexes in media at 37ºC under 5% CO_2_ were brought to the confocal microscope and added to neighboring slide wells immediately following removal of growth media. Imaging was begun immediately after the autofocus was set at about 5 minutes and alternate imaging of paired wells was continued for at least 60 min. Movies are set for simultaneous viewing of each 650-CGH36 ± TAT-CaM for 60 min. Initiate by starting slide presentation.
(PPTX)
