# Supplementary material for: Utilization of cell-penetrating peptide adaptors to enhance delivery of variably charged protein cargos
Source: PLoS One. 2026 Jul 10;21(7):e0345530. doi: 10.1371/journal.pone.0345530 (PMC13354093; doi:10.1371/journal.pone.0345530)
Supplement: S1 Fig — Supercharged GFP sequences were taken from Thompson et al (2012) (Reference #16). (PDF) [file pone.0345530.s001.pdf]

S1 Fig. SEQUENCES AND CHARACTERISTICS OF GFP CARGOS

Supercharged GFP sequences were taken from Thompson et al (2012), *Chemistry & Biology* 29(7) 832-43 (Reference #16), e.g. +9 GFP:

MGHHHHHHGGASKGEELFTGVVPILVELDGDVNGHKFSVRGEGEGDATNGKLTLKFICTTG
KLPVPWPTLVTTLTYGVQCFSRYPDHMKRHDFFKSAMPKGYVQERTISFKKDGKYKTRAEV
KFEGRTLVNRIKLKGRDFKEKGNILGHKLRYNFNSHKVYITADKQKNGIKANFKIRHNVEDGS
VQLADHYQQNTPIGDGPVLLPDNHYLSTQSALSKDPNEKRDHMVLLEFVTAAGITHGMDEL
YK

Sequences for cargos described in text are below:

Purple = vector-encoded (pET19b) His tag

Red = calmodulin binding site

Yellow highlight: HiBiT sequence (see Teo, et al (2021) *Nat. Comm.* 12(1):3721 (Reference #20); GSSG was also included in their HiBiT.

Numbers in parentheses are molecular weight, net charge at pH 7.0 of the entire protein, though they are named for the charge of the GFP.

GFP cargos with C-terminal calmodulin binding sites (CBS)

GFP9-CBS-HiBiT (35.2 KDa, +19)

**MGHHHHHHHHHHSSGHIDDDDKHMSKGEELFTGVVPILVELDGDVNGHKFSVRGEGEG
DATNGKLTLKFICTTGKLPVPWPTLVTTLTYGVQCFSRYPDHMKRHDFFKSAMPKGYVQE
RTISFKKDGKYKTRAEVKFEGRTLVNRIKLKGRDFKEKGNILGHKLRYNFNSHKVYITADKQ
KNGIKANFKIRHNVEDGSVQLADHYQQNTPIGDGPVLLPDNHYLSTQSALSKDPNEKRDH
MVLLEFVTAAGITHGMDELYKGRGSKRRWKKNFIAVSAANRFKKISSSGALLVPRGSVSG
WRLFKKISGGSG**

GFP15-CBS-HiBiT (35.3 KDa, +25)

**MGHHHHHHHHHHSSGHIDDDDKHMSKGERLFTGVVPILVELDGDVNGHKFSVRGEGE
GDATRGKLTLKFICTTGKLPVPWPTLVTTLTYGVQCFSRYPKHMKRHDFFKSAMPEGYVQ
ERTISFKKDGTYKTRAEVKFEGRTLVNRIELKGRDFKEKGNILGHKLEYNFNSHNVYITADKR
KNGIKANFKIRHNVKDGSVQLADHYQQNTPIGRGPVLLPRNHYLSTRSALSKDPKEKRDH**

MVLLEFVTAAGITHGMDELYKGRGSKRRWKKNFIAVSAANRFKKISSSGALLVPRGSVSG
WRLFKKISGGSG

GFP20-CBS-HiBiT (35.5 KDa with tag, + 30 at pH 7.0)

MGHHHHHHHHHHSSGHIDDDDKHMASKGERLFRGKVPILVELKGDVNGHKFSVRGKG
KGDATRGKLTLKFICTTGKLPVPWPTLVTTLTYGVQCFSRYPKHMKQHDFFKSAMPEGYV
QERTISFKDDGTYKTRAEVKFEGDTLVNRIELKGIDFKEDGNILGHKLEYNFNSHNVYITAD
KRKNGIKAKFKIRHNVKDGSVQLADHYQQNTPIGRGPVLLPRNHYLSTRSKLSKDPKEKRD
HMVLLEFVTAAGIKHGRDERYKGRGSKRRWKKNFIAVSAANRFKKISSSGALLVPRGSVS
GWRLFKKISGGSG

GFP25-CBS-HiBiT (35.4 KDa with tag, +35 at pH 7.0)

MGHHHHHHHHHHSSGHIDDDDKHMASKGERLFTGVVPILVELDGDVNGHKFSVRGKG
KGDATRGKLTLKFICTTGKLPVPWPTLVTTLTYGVQCFSRYPKHMKRHDFFKSAMPKGYV
QERTISFKKDGTYKTRAEVKFEGRTLVNRIKLKGRDFKEKGNILGHKLRYNFNSHNVYITAD
KRKNGIKANFKIRHNVKDGSVQLADHYQQNTPIGRGPVLLPRNHYLSTRSALSKDPKEKR
DHMVLLEFVTAAGITHGMDELYKGRGSKRRWKKNFIAVSAANRFKKISSSGALLVPRGSV
SGWRLFKKISGGSG

GFP cargos with N-terminal calmodulin binding sites (CBS)

CBS-GFP9-HiBiT (303aa, 34275.02 Da with tag, + 17 at pH 7.0)

MGHHHHHHHHHHSSGHIDDDDKHMKRRWKKNFIAVSAANRFKKISSSGALASKGEELF TGVVPILVELDGDVNGHKFSVRGEGEGDATNGKLTLKFICTTGKLPVPWPTLVTTLTYGVQ CFSRYPDHMKRHDFFKSAMPKGYVQERTISFKKDGKYKTRAEVKFEGRTLVNRIKLKGRD FKEKGNILGHKLRYNFNSHKVYITADKQKNGIKANFKIRHNVEDGSVQLADHYQQNTPIG DGPVLLPDNHYLSTQSALSKDPNEKRDHMVLLEFVTAAGITHGMDELYKVSGWRLFKKIS GGSG

CBS-GFP15-HiBiT (303aa, 34442.37 Da with tag, + 23 at pH 7.0)

MGHHHHHHHHHHSSGHIDDDDKHMKRRWKKNFIAVSAANRFKKISSSGALASKGERLF TGVVPILVELDGDVNGHKFSVRGEGEGDATRGKLTLKFICTTGKLPVPWPTLVTTLTYGVQ CFSRYPKHMKRHDFFKSAMPEGYVQERTISFKKDGTYKTRAEVKFEGRTLVNRIELKGRDF KEKGNILGHKLEYNFNSHNVYITADKRKNGIKANFKIRHNVKDGSVQLADHYQQNTPIGR GPVLLPRNHYLSTRSALSKDPKEKRDHMVLLEFVTAAGITHGMDELYKVSGWRLFKKISGG SG

CBS-GFP20-HiBiT (303aa, 34565.6 Da with tag, +28 at pH 7.0)

MGHHHHHHHHHHSSGHIDDDDKHMKRRWKKNFIAVSAANRFKKISSSGALASKGERLF RGKVPILVELKGDVNGHKFSVRGKGKGDATRGKLTLKFICTTGKLPVPWPTLVTTLTYGVQ CFSRYPKHMKQHDFFKSAMPEGYVQERTISFKDDGTYKTRAEVKFEGDTLVNRIELKGIDF KEDGNILGHKLEYNFNSHNVYITADKRKNGIKAKFKIRHNVKDGSVQLADHYQQNTPIGR GPVLLPRNHYLSTRSKLSKDPKEKRDHMVLLEFVTAAGIKHGRDERYKVSGWRLFKKISGG SG

CBS-GFP25-HiBiT (303aa, 34465.68 Da with tag, +33 at pH 7.0)

MGHHHHHHHHHHSSGHIDDDDKHMKRRWKKNFIAVSAANRFKKISSSGALASKGERLF TGVVPILVELDGDVNGHKFSVRGKGKGDATRGKLTLKFICTTGKLPVPWPTLVTTLTYGVQ CFSRYPKHMKRHDFFKSAMPKGYVQERTISFKKDGTYKTRAEVKFEGRTLVNRIKLKGRDF KEKGNILGHKLRYNFNSHNVYITADKRKNGIKANFKIRHNVKDGSVQLADHYQQNTPIGR

GPVLLPRNHYLSTRSALSKDPKEKRDHMVLLEFVTAAGITHGMDELYKVSGWRLFKKISGG
SG

CBS-GFP36-HiBiT (303aa, 34770.28 Da with tag, +44 at pH 7.0)

MGHHHHHHHHHHSSGHIDDDDKHMKRRWKKNFIAVSAANRFKKISSSGALASKGERLF
RGKVPILVELKGDVNGHKFSVRGKGKGDATRGKLTLKFICTTGKLPVPWPTLVTTLTYGVQ
CFSRYPKHMKRHDFFKSAMPKGYVQERTISFKKDGKYKTRAEVKFEGRTLVNRIKLKGRDF
KEKGNILGHKLRYNFNSHKVYITADKRKNGIKAKFKIRHNVKDGSVQLADHYQQNTPIGR
GPVLLPRNHYLSTRSKLSKDPKEKRDHMVLLEFVTAAGIKHGRDERYKVSGWRLFKKISGG
SG
