# Supplementary figures and images for: Utilization of cell-penetrating peptide adaptors to enhance delivery of variably charged protein cargos
Source: PLoS One. 2026 Jul 10;21(7):e0345530. doi: 10.1371/journal.pone.0345530 (PMC13354093; doi:10.1371/journal.pone.0345530)

# Purified CGH cargos - Dylight 650 Labeled

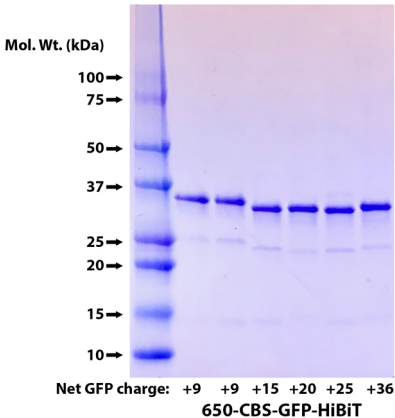

Supplement: S2 Fig — As described in Methods, CGH cargos expressed in E coli were extracted, purified on 5 ml cobalt columns and then again using open phase CaM column matrix. Eluted protein was concentrated, desalted into buffer with 10% glycerol and 1M NaCl and labeled with 0.4 mol Dylight NHS ester per mol of protein. Following dye removal, the cargos were quantitated by Bradford and 2 µg of each was run on a 4–20% precast biorad gel, which was stained by Coomassie and imaged. An unaltered, uncropped photograph of the gel used to make this image is available at https://doi.org/10.6084/m9.figshare.32736102. (PDF) [file pone.0345530.s002.pdf]

**A** 100nM CGHs with 150nM TAT-CaM

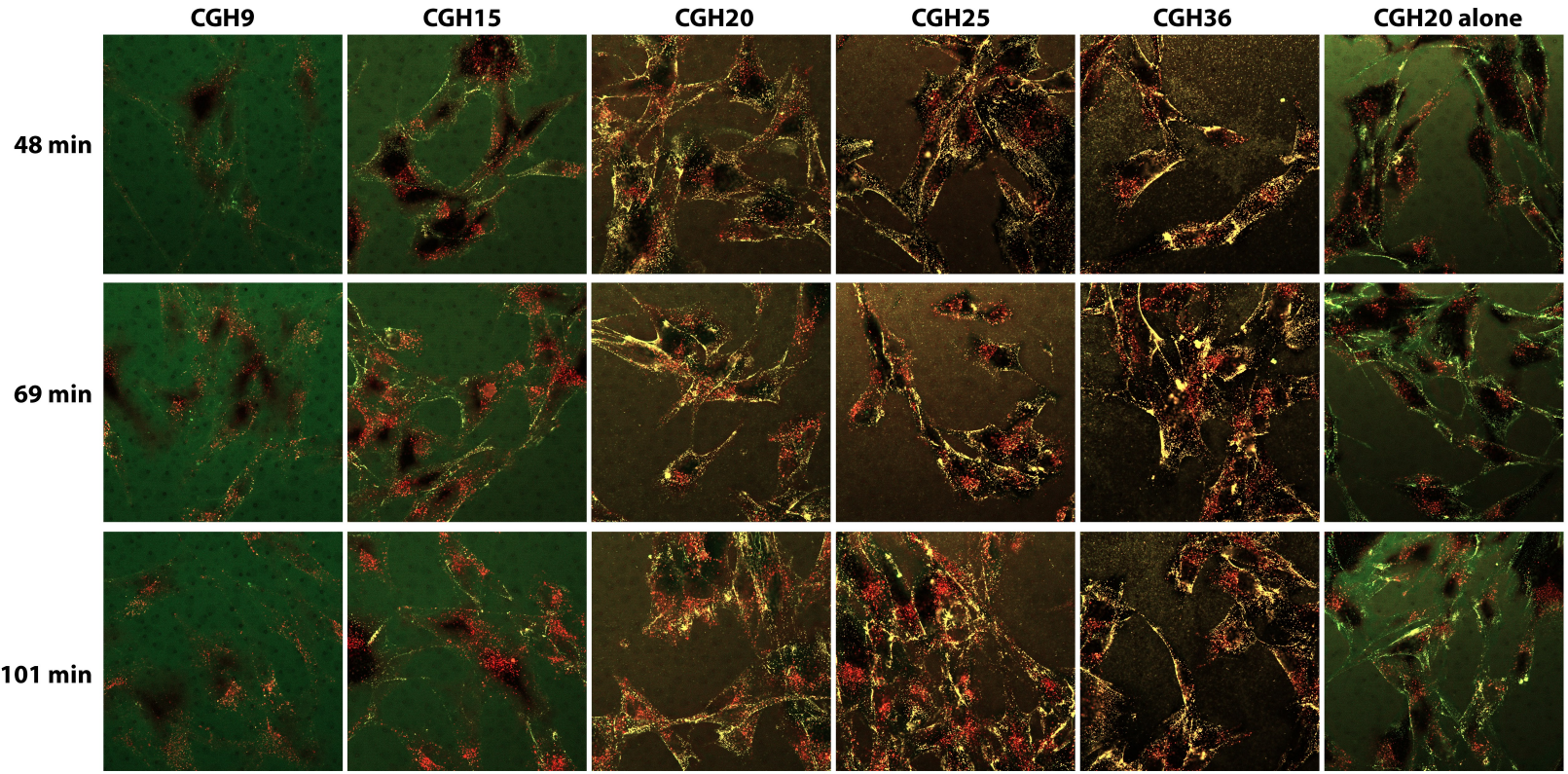

**B** 400 nM CGHs with 600 nM TAT-CaM

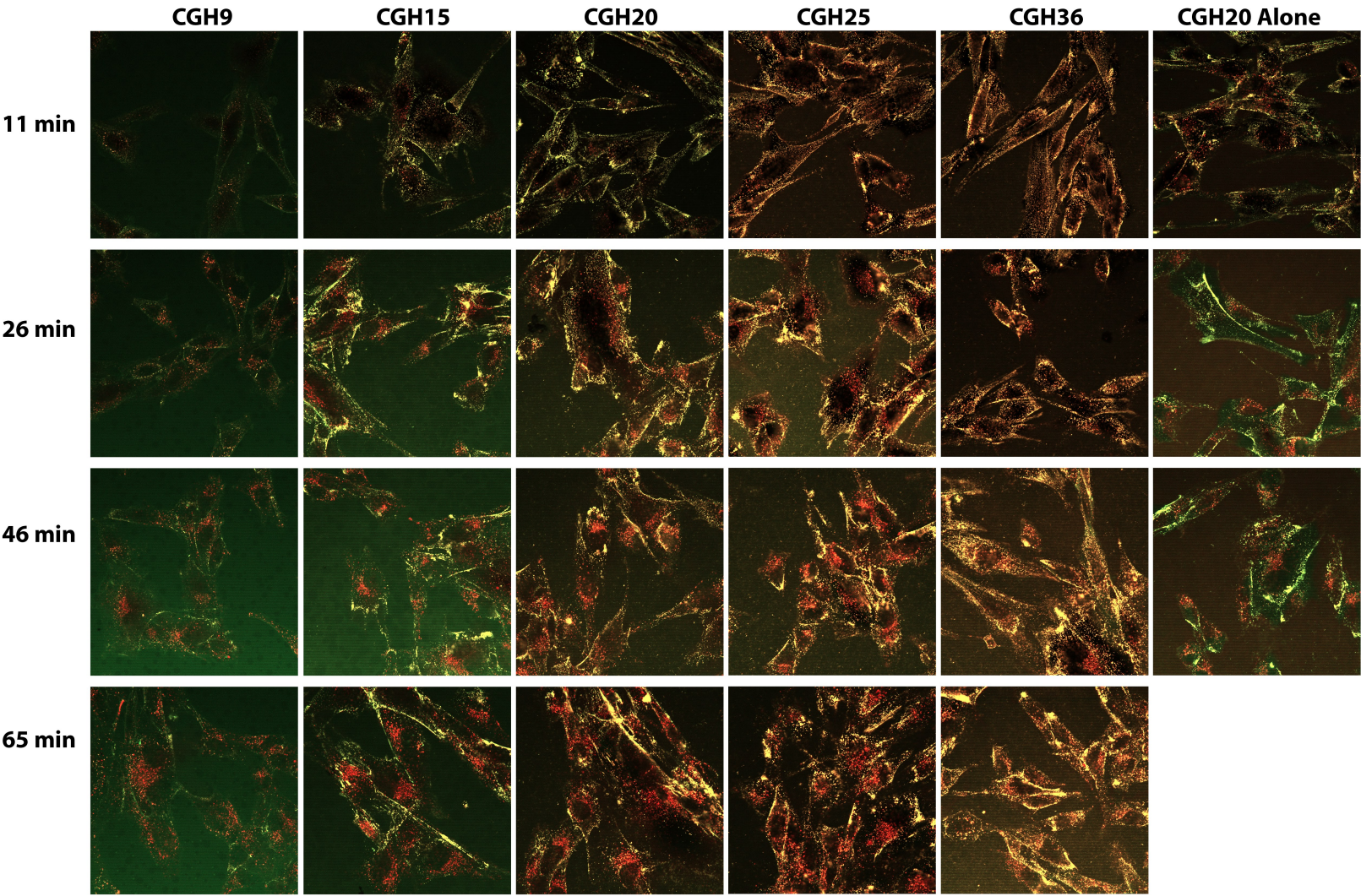

Supplement: S3 Fig — Far-red labeled CGH cargos (CGH9, CGH15, CGH20, CGH25, CGH36) were combined with TAT-CaM, diluted in media and used to treat cells as described in the Methods. One well included only CGH20 as a limited specificity control that is strictly comparable only to internalization of the CGH20/TAT-CaM complex. (A) Time series showing internalization of 100 nM CGH cargos with 150 nM TAT-CaM (n = 3), alongside cells treated with 100 nM CGH20 alone (far right) as a limited control that shows intrinsic internalization at this concentration. Profiles of cargo internalization beginning 48 min, 69 min and 101 min after complex addition (48 min used in Fig 4A). Imaging order was not consistent between profiles. (B) Time series showing internalization of 400 nM CGH cargos with 600 nM TAT-CaM (n = 4) alongside cells treated with 400 nM CGH20 alone as a limited control that shows intrinsic internalization at this concentration (see Fig 1C). Profiles of cargo internalization beginning 11, 26, 46 and 65 min after complex addition (46 min used in Fig 4B). Imaging order: CGH36, CGH25, CGH9, CGH15, CGH20 and CGH20 alone. (PDF) [file pone.0345530.s003.pdf]

**A** 100 nM CGHs with 150 nM TAT-AUR-CaM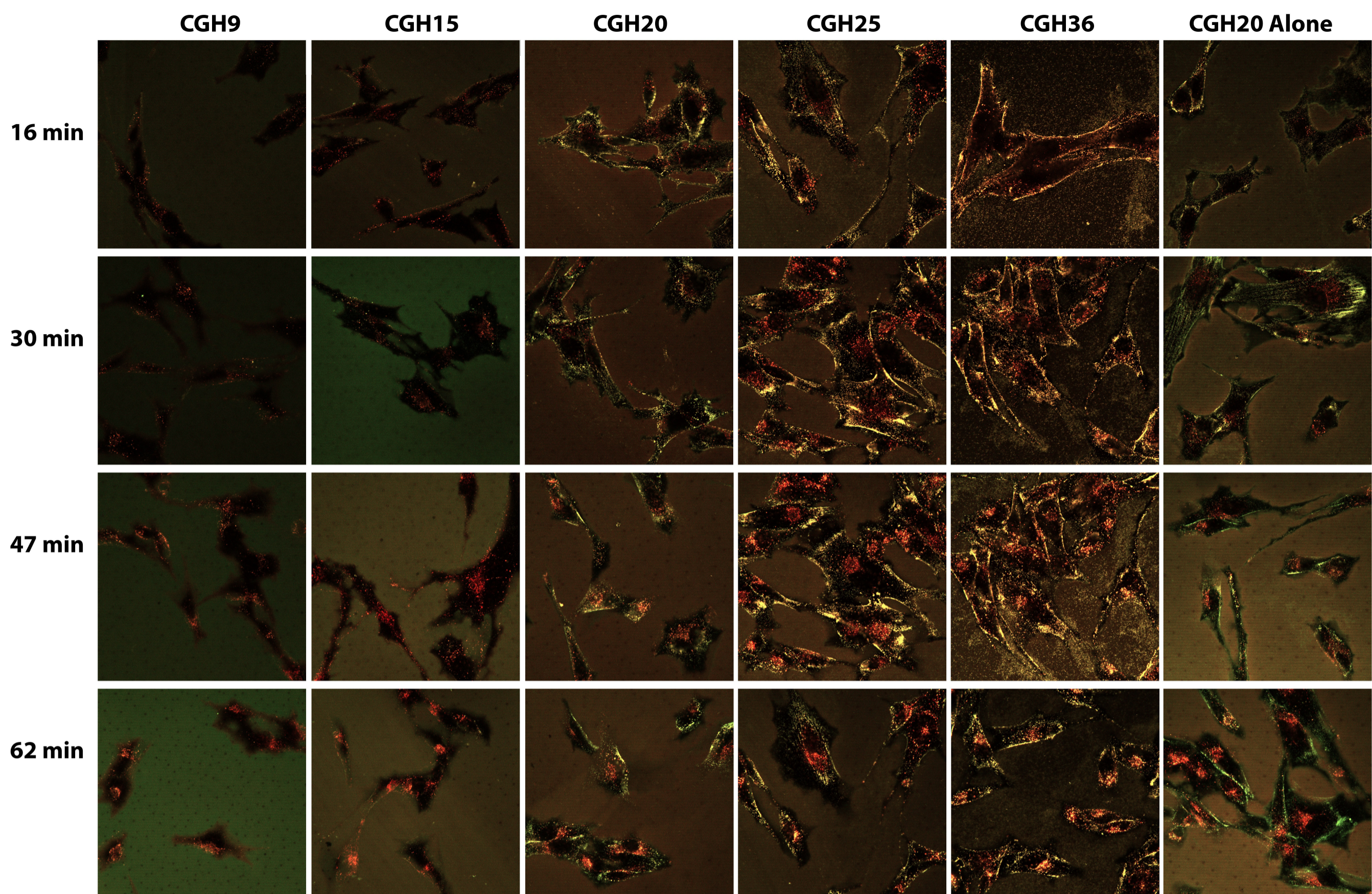**B** 400 nM CGHs with 450 nM TAT-AUR-CaM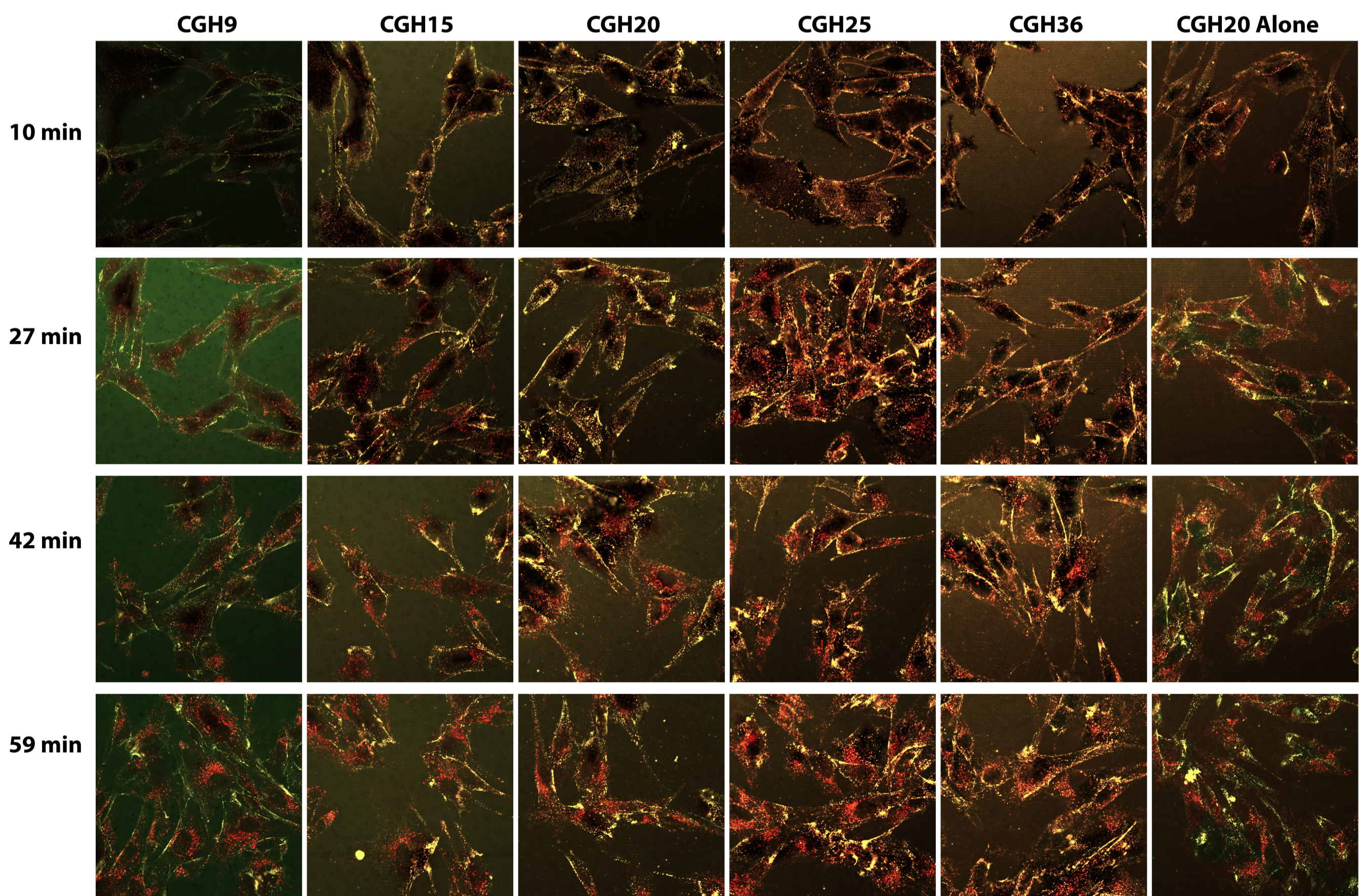

Supplement: S4 Fig — Far-red labeled CGH cargos combined with TAT-AUR-CaM were diluted in media then added to cells as in Methods. (A) Time series showing internalization of 100 nM of CGH cargos with 150 nM TAT-AUR-CaM, alongside cells treated with 100 nM CGH20 alone as a limited specificity control (n = 3). Profiles of cargo internalization beginning 16, 30, 47 and 62 min after complex addition (47 min used in Fig 4C). Imaging order: CGH9, CGH15, CGH20, CGH20 alone, CGH36 and CGH25. (B) Time series showing internalization of 400 nM CGH cargos with 600 nM TAT-AUR-CaM, alongside cells treated with 400 nM CGH20 alone as a limited specificity control (n = 3). Profiles of cargo internalization beginning 10, 27, 42 and 59 min after complex addition (42 min used in Fig 4D). Imaging order: CGH36, CGH25, CGH9, CGH15, CGH20 and CGH20 alone. (PDF) [file pone.0345530.s004.pdf]

**A** 100 nM Cargos with 150 nM TAT-LAH4-CaM

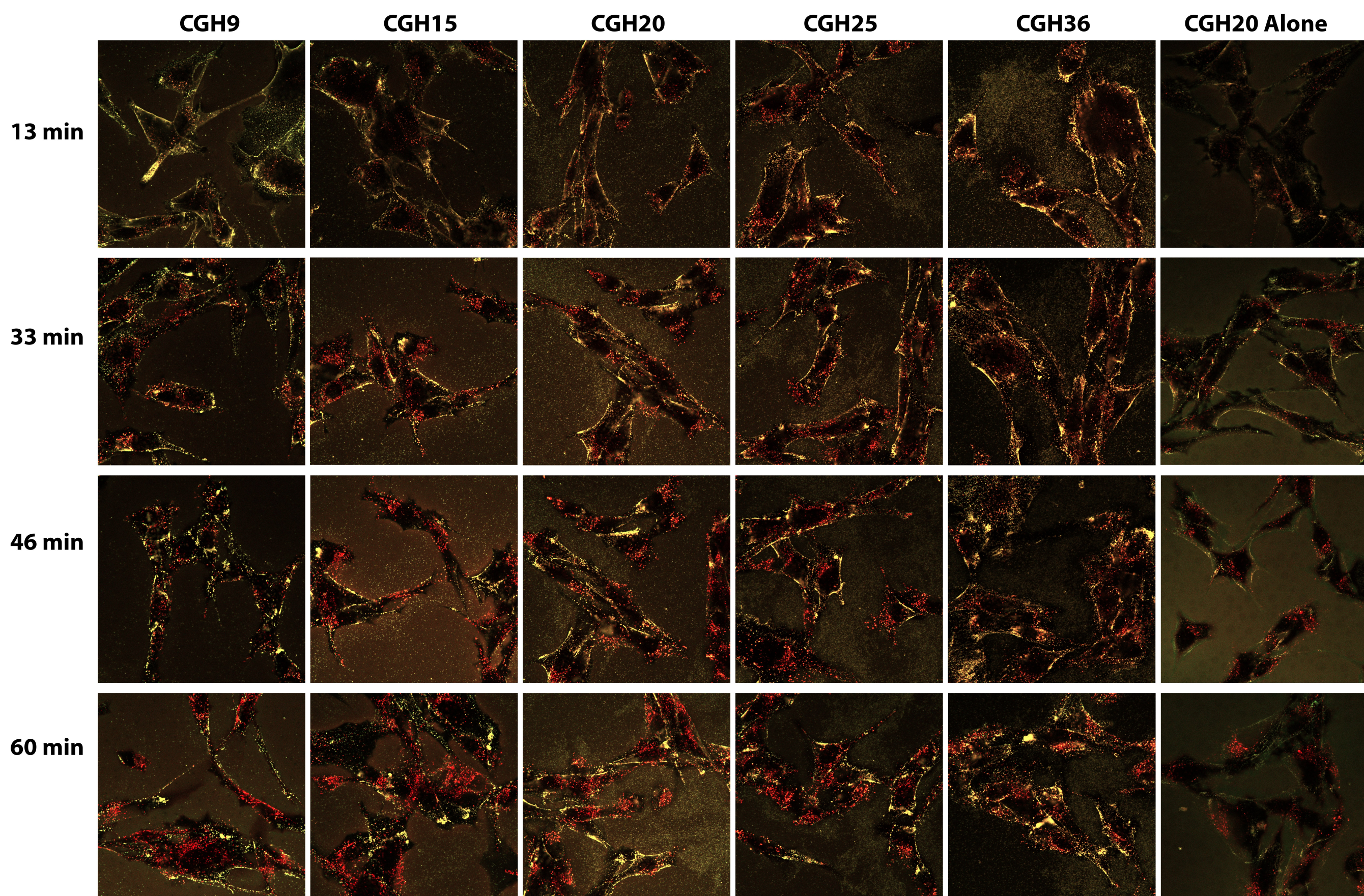

**B** 400 nM Cargos with 600 nM TAT-LAH4-CaM

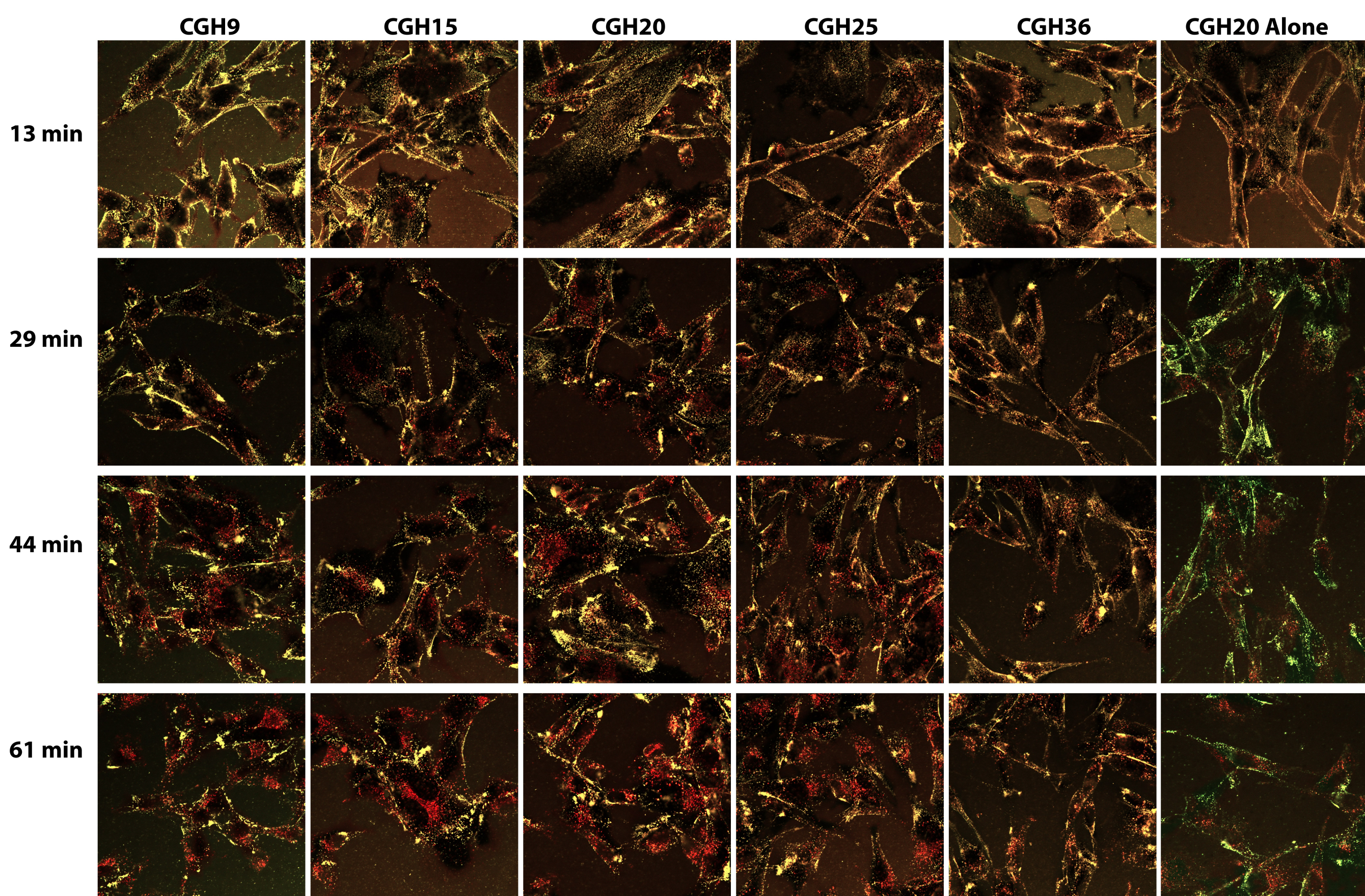

Supplement: S5 Fig — Cells were treated with complexes of the CGH cargos and TAT-LAH4-CaM, along with a limited specificity control containing CGH20 alone. (A) Time series showing internalization of 100 nM of CGH cargo with 150 nM TAT-LAH4-CaM, alongside a control with 100 nM CGH20 alone (n = 3). Profiles of cargo internalization beginning 13, 33, 46 and 60 min after complex addition (46 min used in Fig 5A). Imaging order: CGH9, CGH15, CGH20, CGH20 alone, CGH36 and CGH25. (B) Time series showing internalization of 400 nM CGH cargos with 600 nM TAT-LAH4-CaM, alongside a control with 400 nM CGH20 alone (n = 3). Profiles of cargo internalization beginning 13, 29, 44 and 61 min after complex addition (44 min used in Fig 5B). Imaging order: CGH9, CGH15, CGH36, CGH25, CGH20 and CGH20 alone. (PDF) [file pone.0345530.s005.pdf]

**A** 100 nM Cargos with 110 nM GFP-CaM

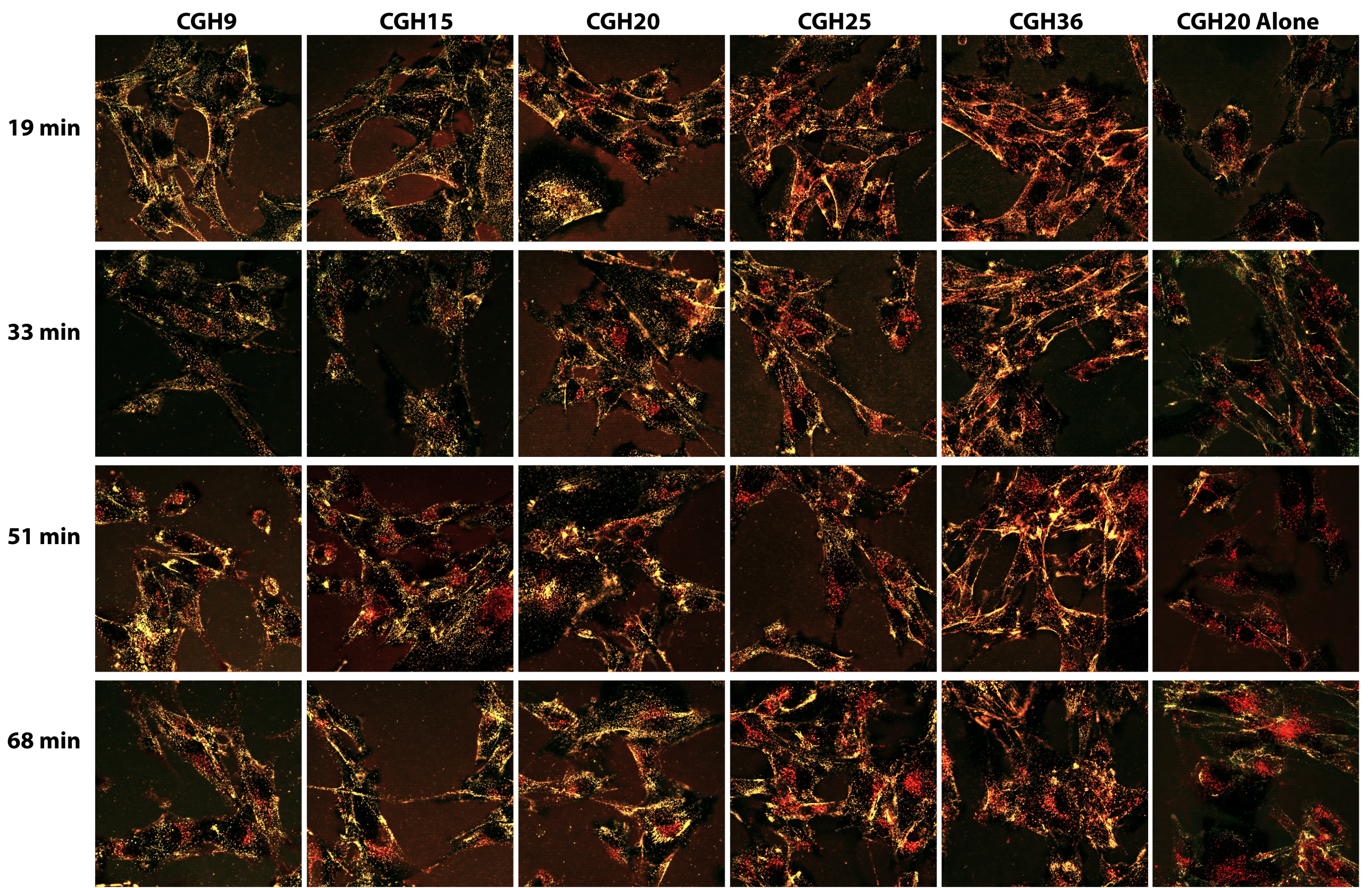

**B** 400 nM Cargos with 440 nM GFP-CaM

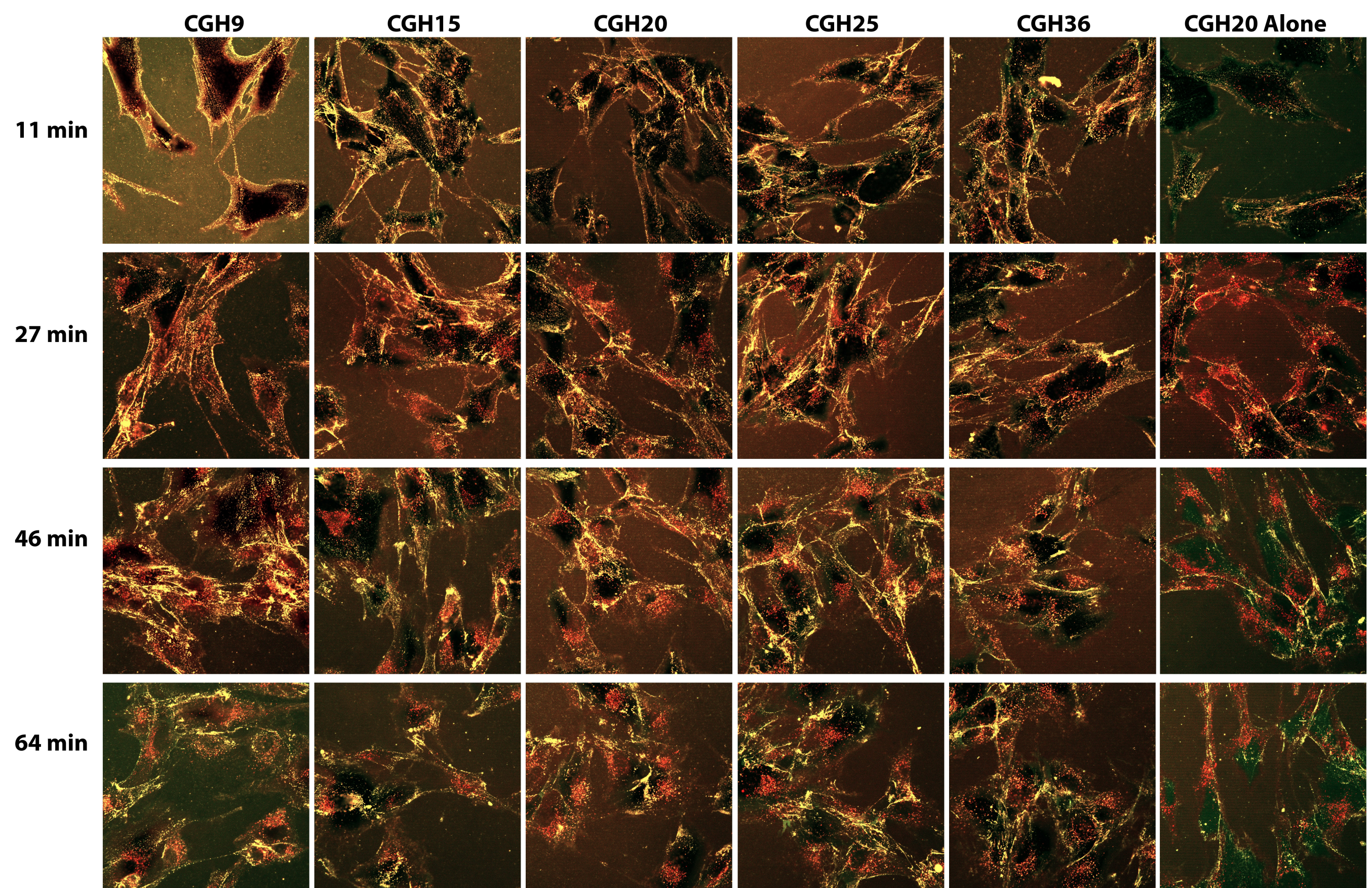

Supplement: S6 Fig — Cells were treated with complexes of the CGH cargos and GFP-CaM, along with a limited specificity control containing CGH20 alone. (A) Time series showing internalization of 100 nM of CGH cargo with 110 nM GFP-CaM, alongside a control with 100 nM CGH20 alone (n = 3). Profiles of cargo internalization beginning 10, 33, 46 and 68 min after complex addition (46 min used in Fig 5C). (B) Time series showing internalization of 400 nM CGH cargos with 440 nM GFP-CaM, alongside a control with 400 nM CGH20 alone (n = 4). Profiles of cargo internalization beginning 11, 27, 44 and 64 min after complex addition (44 min used in Fig 5D). Imaging order: CGH9, CGH15, CGH20, CGH20 alone, CGH36 and CGH25. (PDF) [file pone.0345530.s006.pdf]

**A** 100 nM Cargos with 110 nM TAT-NMR-CaM

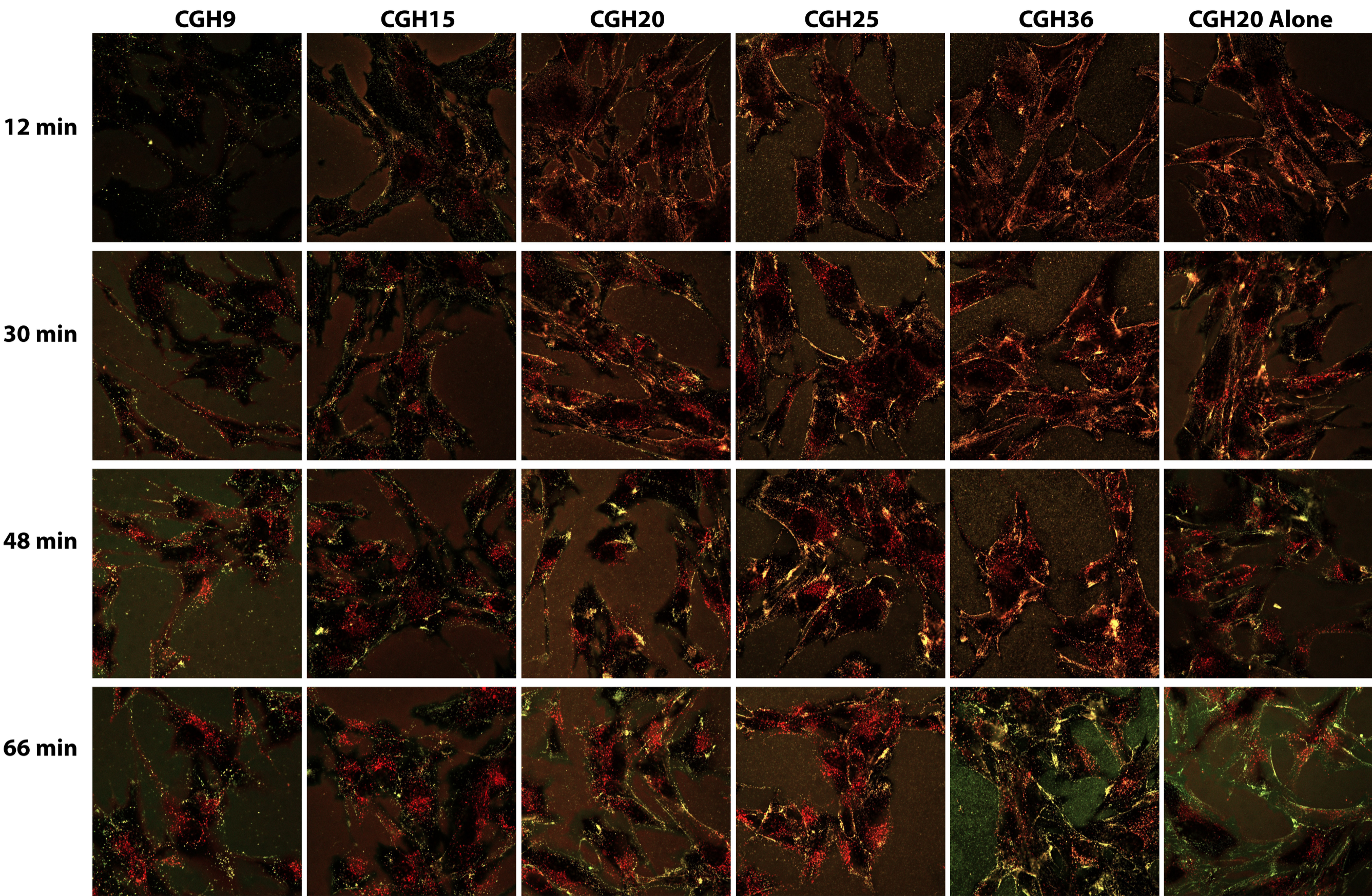

**B** 400 nM Cargos with 440 nM TAT-NMR-CaM

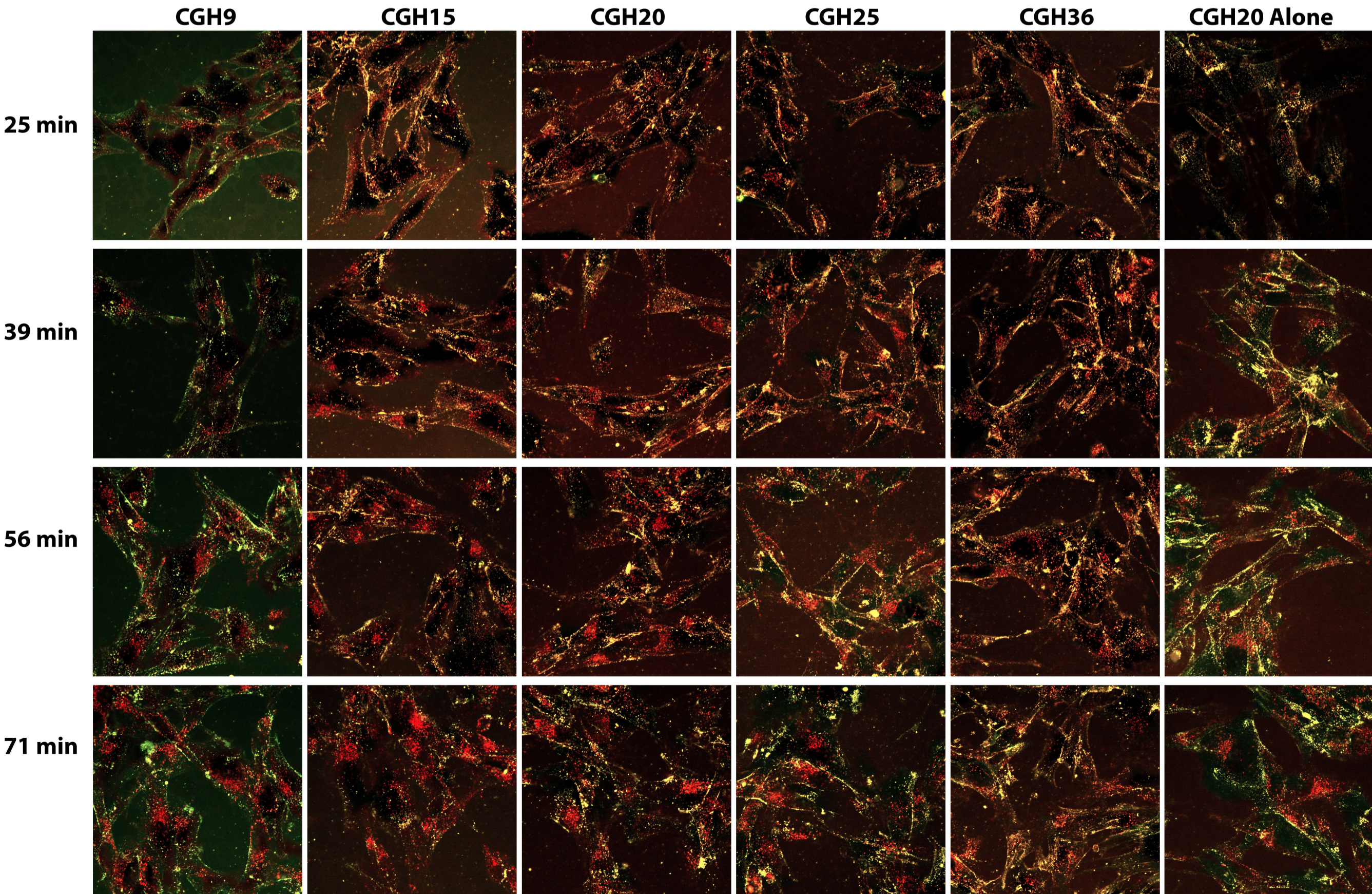

Supplement: S7 Fig — Cells were treated with complexes of the CGH cargos and TAT-NMR-CaM, along with a limited specificity control containing CGH20 alone. (A) Cells treated with 100 nM of CGH cargo with 110 nM TAT-NMR-CaM, alongside a control with 100 nM CGH20 alone (n = 5). Profiles of cargo internalization beginning 12, 30, 46 and 59 min after complex addition (46 min used in Fig 5E). (B) Cells treated with 400 nM CGH cargos plus 440 nM TAT-NMR-CaM alongside a control with 400 nM CGH20 alone (n = 4). Profiles of cargo internalization beginning 10, 27, 44 and 66 min after complex addition (44 min used in Fig 5F). Imaging order: CGH9, CGH15, CGH20, CGH20 alone, CGH36 and CGH25. (PDF) [file pone.0345530.s007.pdf]

100 nM CGH15 alone and with excess of 5 adapters

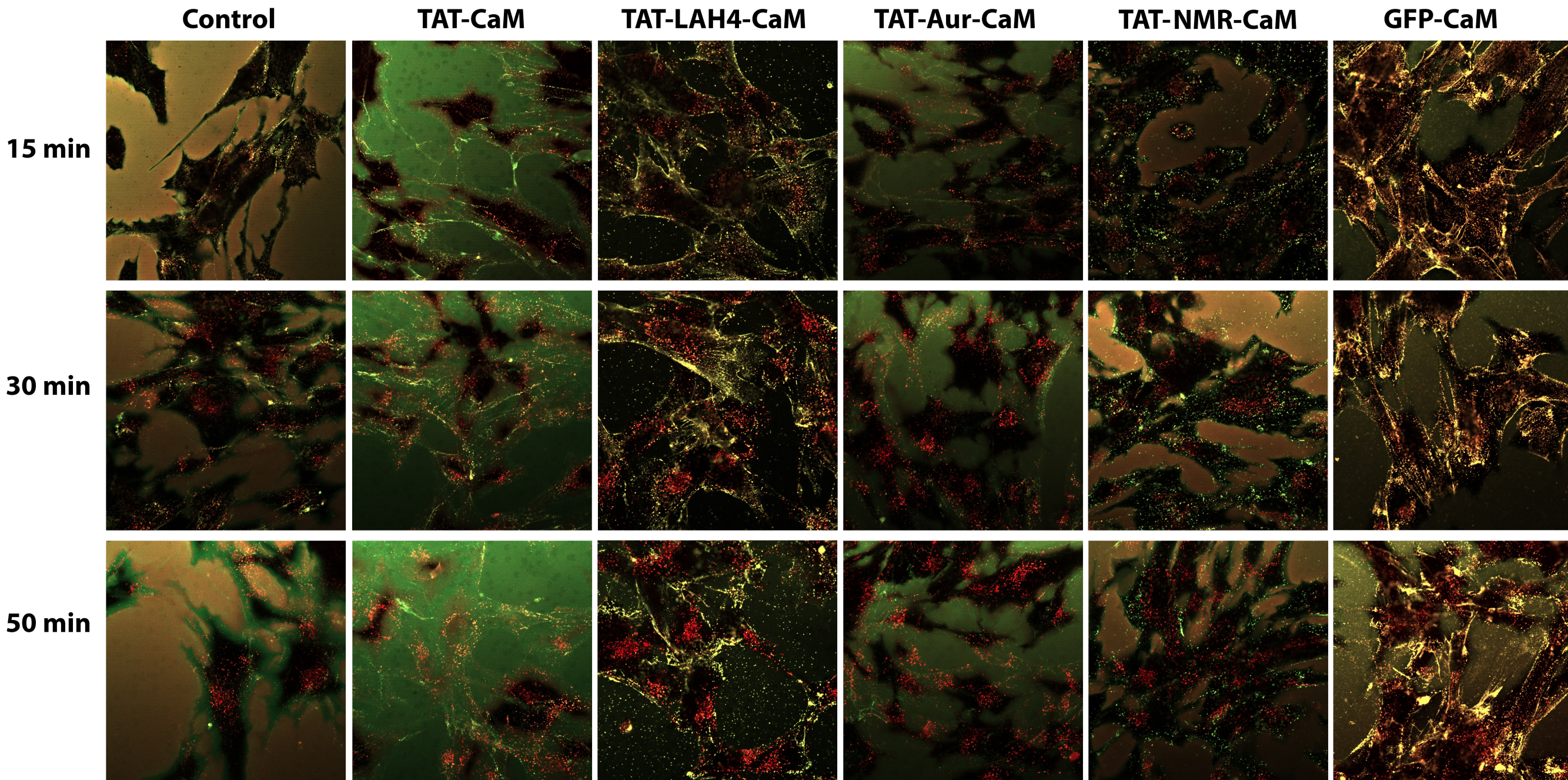

Supplement: S9 Fig — Internalization of 100 nM CGH15 alone or with 150 nM TAT-CaM, TAT-LAH4-CaM, TAT-AUR-CaM, TAT-NMR-CaM and GFP-CaM. Representative experiment showing profiles of cargo internalization imaged beginning 15, 30 and 50 min after complex addition (50 min used in Fig 6B). (n = 3). (PDF) [file pone.0345530.s009.pdf]

# 100 nM CGH20 alone and with excess of 5 adapters

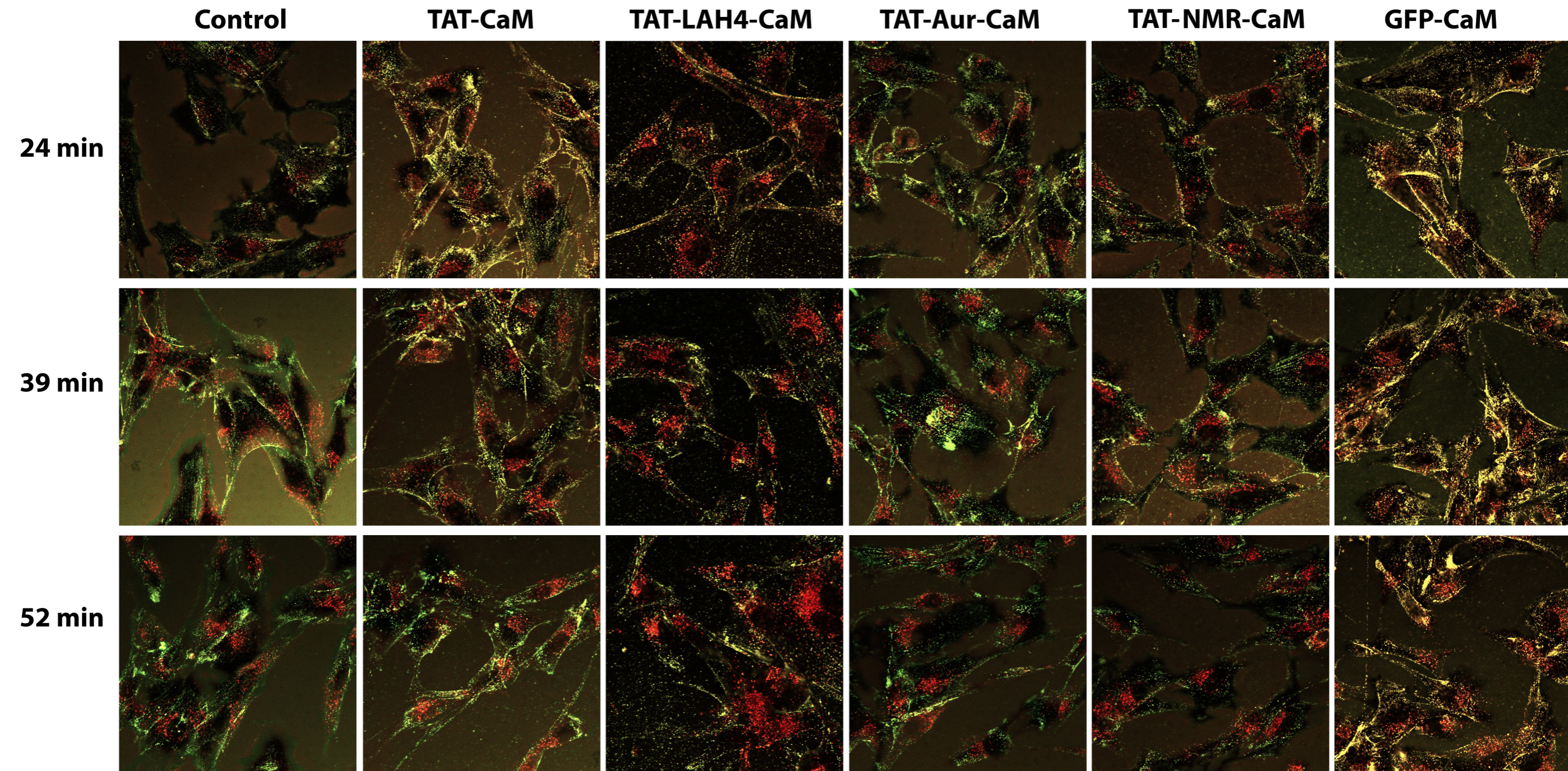

Supplement: S10 Fig — Internalization of 100 nM CGH20 alone or with 150 nM TAT-CaM, TAT-LAH4-CaM, TAT-AUR-CaM, TAT-NMR-CaM and GFP-CaM. Representative experiment showing profiles of cargo internalization imaged beginning 24, 39 and 52 min after complex addition (52 min used in Fig 6C). (n = 4). (PDF) [file pone.0345530.s010.pdf]

## Slide 1
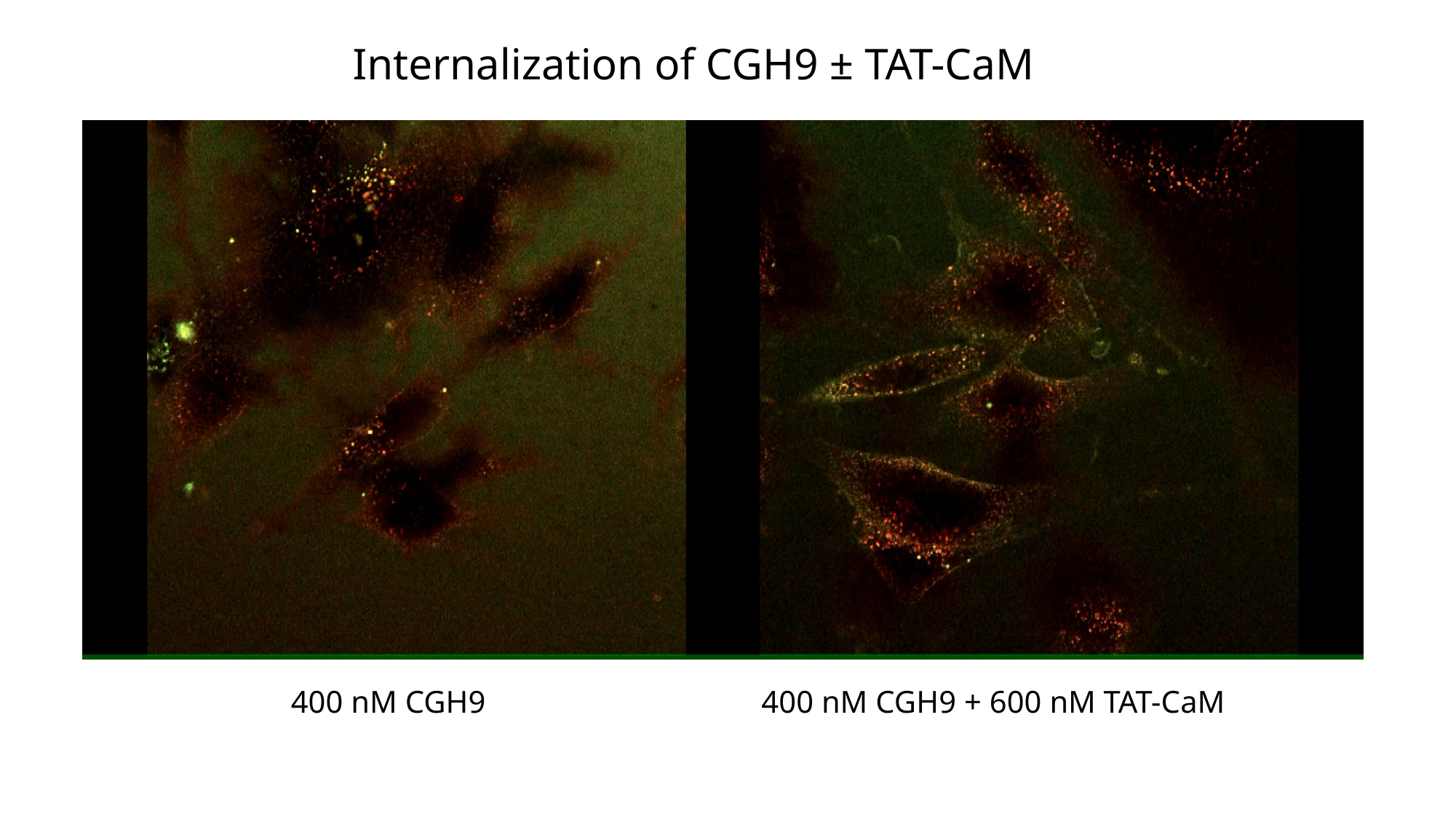

Internalization of CGH9 ± TAT-CaM
400 nM CGH9
400 nM CGH9 + 600 nM TAT-CaM

Supplement: S13 Fig — 650-CGH9/TAT-CaM complexes were set up and diluted in media as in other experiments. Complexes in media at 37ºC under 5% CO2 were brought to the confocal microscope and added to neighboring slide wells immediately following removal of growth media. Imaging was begun immediately after the autofocus was set at about 5 minutes and alternate imaging of paired wells was continued for at least 60 min. Movies are set for simultaneous viewing of each 650-CGH9 ± TAT-CaM for 60 min. Initiate by starting slide presentation. (PPTX) [file pone.0345530.s013.pptx]

## Slide 1
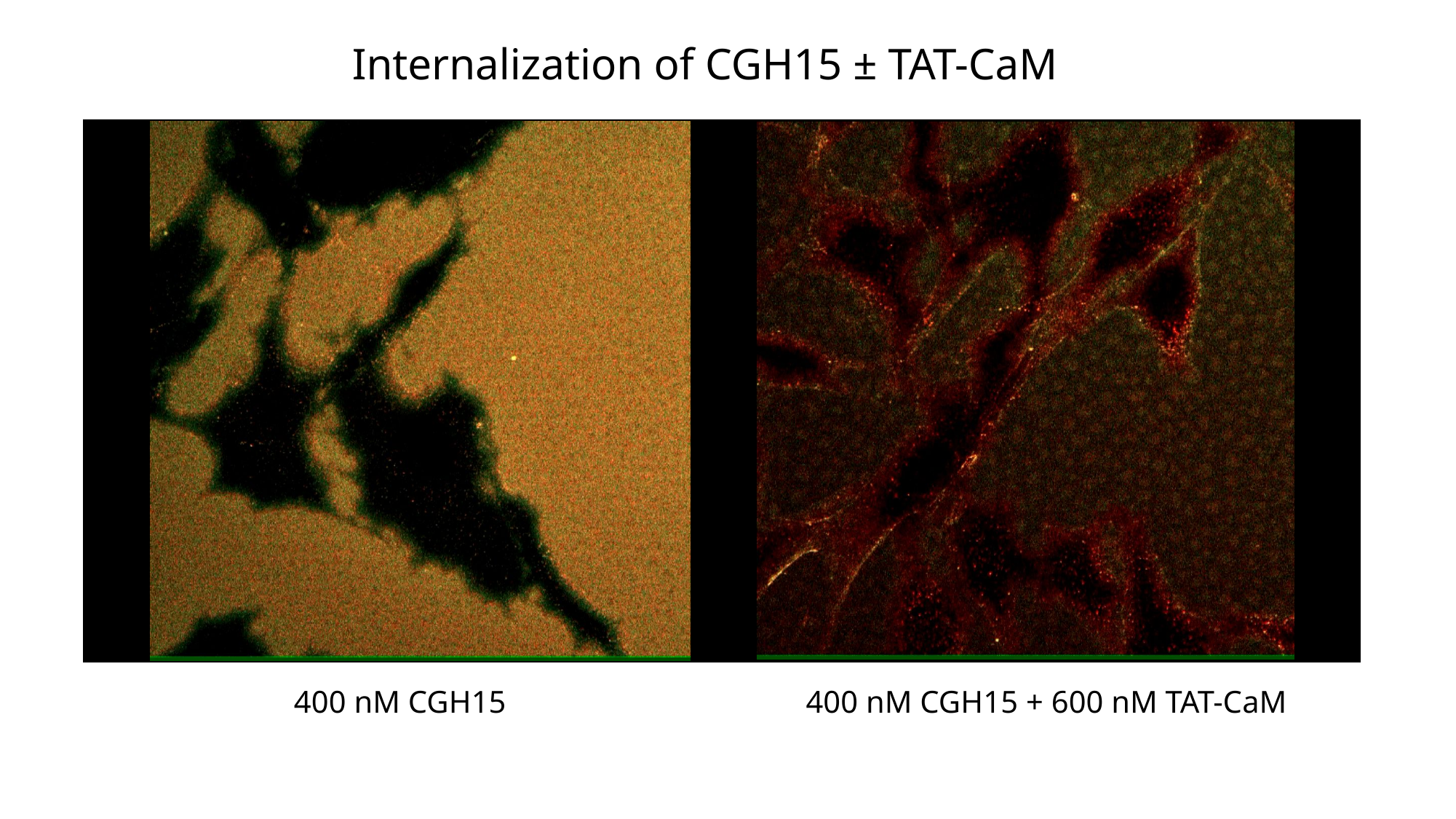

Internalization of CGH15 ± TAT-CaM
400 nM CGH15
400 nM CGH15 + 600 nM TAT-CaM

Supplement: S14 Fig — 650-CGH15/TAT-CaM complexes were set up and diluted in media as in other experiments. Complexes in media at 37ºC under 5% CO2 were brought to the confocal microscope and added to neighboring slide wells immediately following removal of growth media. Imaging was begun immediately after the autofocus was set at about 5 minutes and alternate imaging of paired wells was continued for at least 60 min. Movies are set for simultaneous viewing of each 650-CGH15 ± TAT-CaM for 60 min. Initiate by starting slide presentation. (PPTX) [file pone.0345530.s014.pptx]

## Slide 1
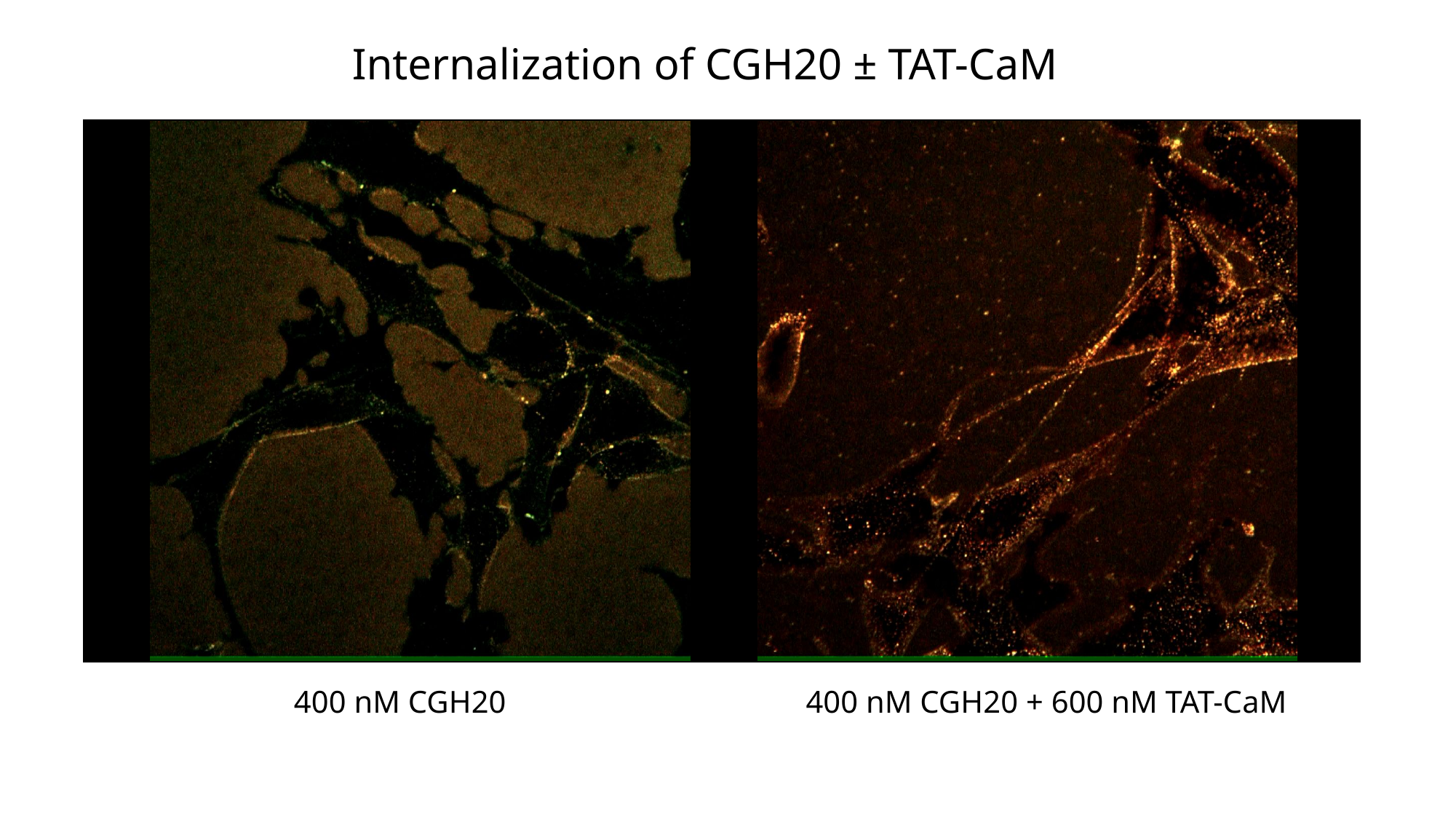

Internalization of CGH20 ± TAT-CaM
400 nM CGH20
400 nM CGH20 + 600 nM TAT-CaM

Supplement: S15 Fig — 650-CGH20/TAT-CaM complex were set up and diluted in media as in other experiments. Complexes in media at 37ºC under 5% CO2 were brought to the confocal microscope and added to neighboring slide wells immediately following removal of growth media. Imaging was begun immediately after the autofocus was set at about 5 minutes and alternate imaging of paired wells was continued for at least 60 min. Movies are set for simultaneous viewing of each 650-CGH20 ± TAT-CaM for 60 min. Initiate by starting slide presentation. (PPTX) [file pone.0345530.s015.pptx]

## Slide 1
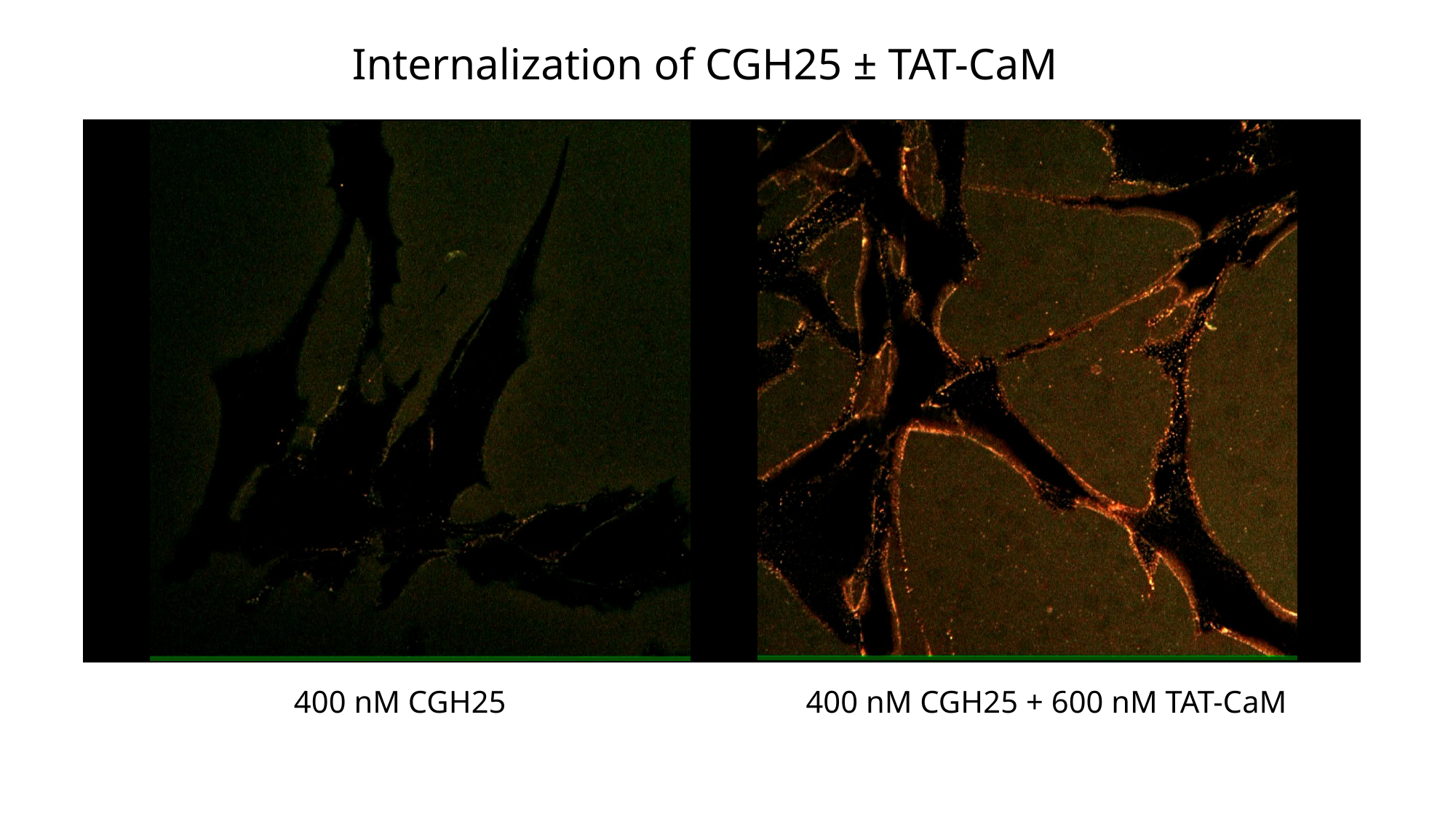

Internalization of CGH25 ± TAT-CaM
400 nM CGH25
400 nM CGH25 + 600 nM TAT-CaM

Supplement: S16 Fig — 650-CGH25/TAT-CaM complexes were set up and diluted in media as in other experiments. Complexes in media at 37ºC under 5% CO2 were brought to the confocal microscope and added to neighboring slide wells immediately following removal of growth media. Imaging was begun immediately after the autofocus was set at about 5 minutes and alternate imaging of paired wells was continued for at least 60 min. Movies are set for simultaneous viewing of each 650-CGH25 ± TAT-CaM for 60 min. Initiate by starting slide presentation. (PPTX) [file pone.0345530.s016.pptx]

## Slide 1
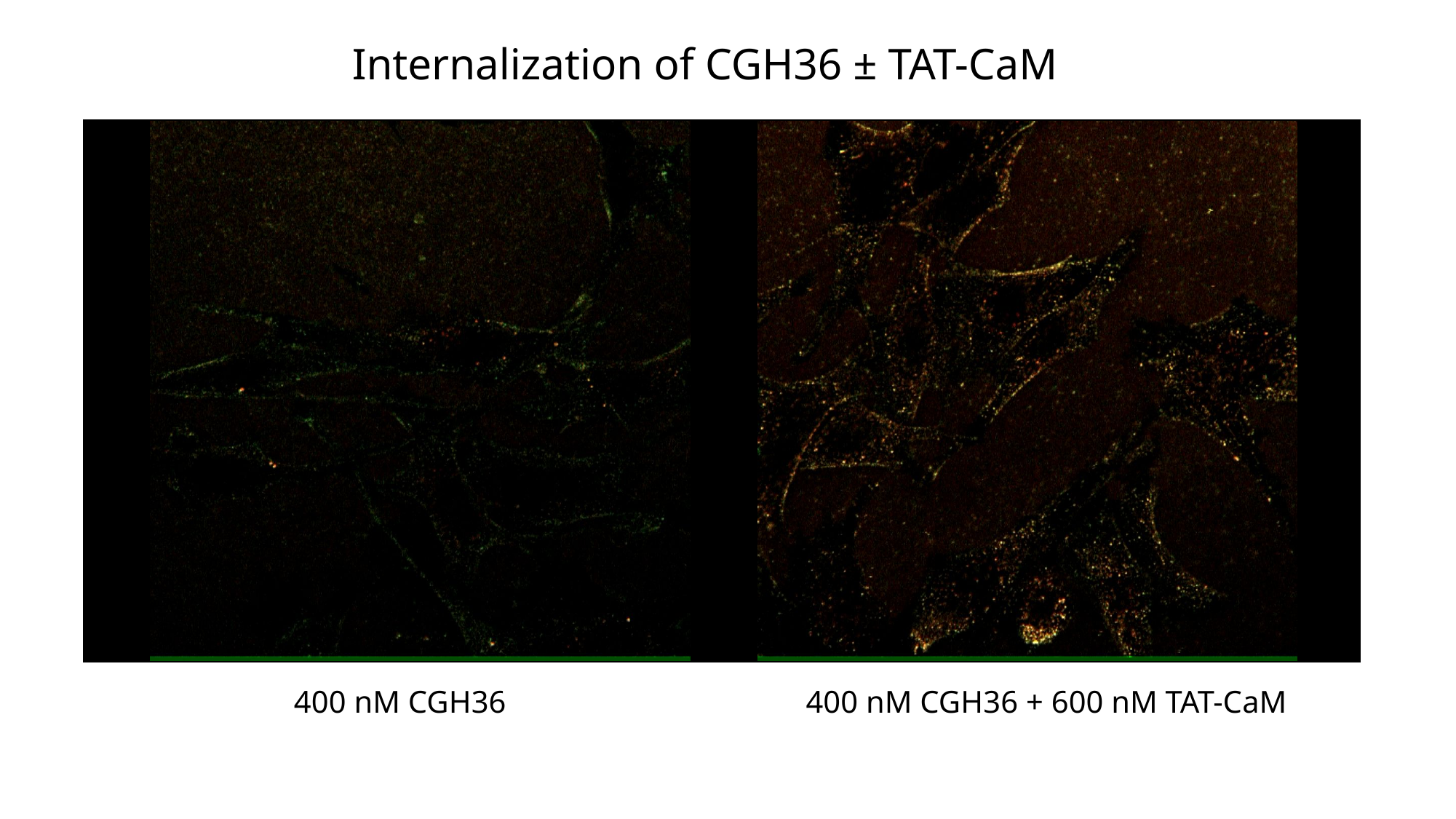

Internalization of CGH36 ± TAT-CaM
400 nM CGH36
400 nM CGH36 + 600 nM TAT-CaM

Supplement: S17 Fig — 650-CGH36/TAT-CaM complexes were set up and diluted in media as in other experiments. Complexes in media at 37ºC under 5% CO2 were brought to the confocal microscope and added to neighboring slide wells immediately following removal of growth media. Imaging was begun immediately after the autofocus was set at about 5 minutes and alternate imaging of paired wells was continued for at least 60 min. Movies are set for simultaneous viewing of each 650-CGH36 ± TAT-CaM for 60 min. Initiate by starting slide presentation. (PPTX) [file pone.0345530.s017.pptx]
