# Supplementary material for: Utilization of cell-penetrating peptide adaptors to enhance delivery of variably charged protein cargos
Source: PLoS One. 2026 Jul 10;21(7):e0345530. doi: 10.1371/journal.pone.0345530 (PMC13354093; doi:10.1371/journal.pone.0345530)
Supplement: S8 Fig — Internalization of 100 nM CGH9 alone or with 250 nM TAT-CaM, TAT-LAH4-CaM and TAT-AUR-CaM or 110nM TAT-NMR-CaM and GFP-CaM. Representative experiment showing profiles of cargo internalization imaged beginning 20, 35 and 47 min after complex addition (47 min used in Fig 6A). (n = 3). (PDF) [file pone.0345530.s008.pdf]

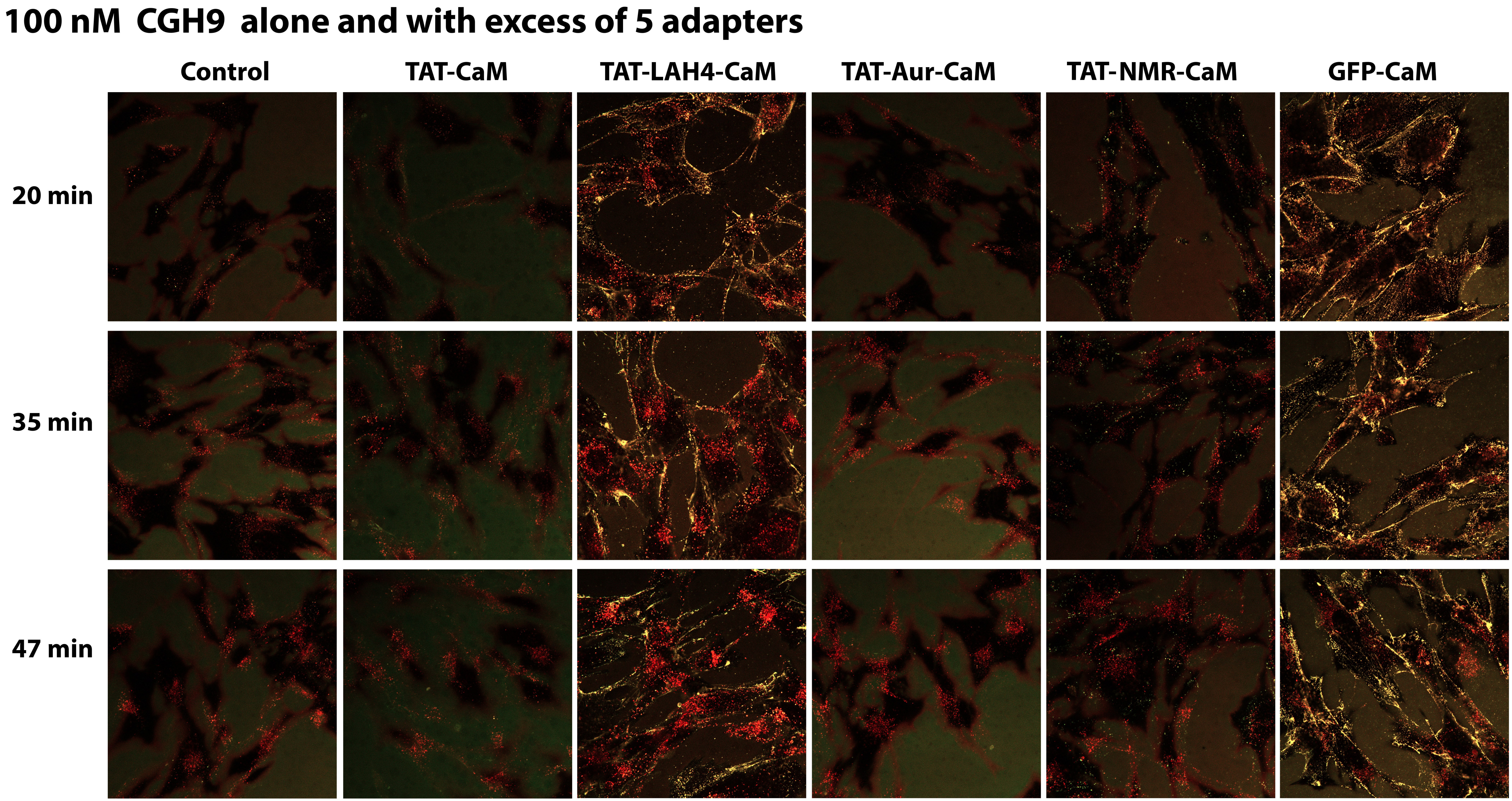
100 nM CGH9 alone and with excess of 5 adapters
Control
TAT-CaM
TAT-LAH4-CaM
TAT-Aur-CaM
TAT-NMR-CaM
GFP-CaM
20 min
35 min
47 min
