# Supplementary material for: Utilization of cell-penetrating peptide adaptors to enhance delivery of variably charged protein cargos
Source: PLoS One. 2026 Jul 10;21(7):e0345530. doi: 10.1371/journal.pone.0345530 (PMC13354093; doi:10.1371/journal.pone.0345530)
Supplement: S11 Fig — Internalization of 100 nM CGH25 alone or with 500 nM TAT-CaM, TAT-LAH4-CaM and TAT-AUR-CaM or 110nM TAT-NMR-CaM and GFP-CaM. Representative experiment showing profiles of cargo internalization imaged beginning 13, 38 and 58 min after complex addition (58 min used in Fig 6D). (n = 4). (PDF) [file pone.0345530.s011.pdf]

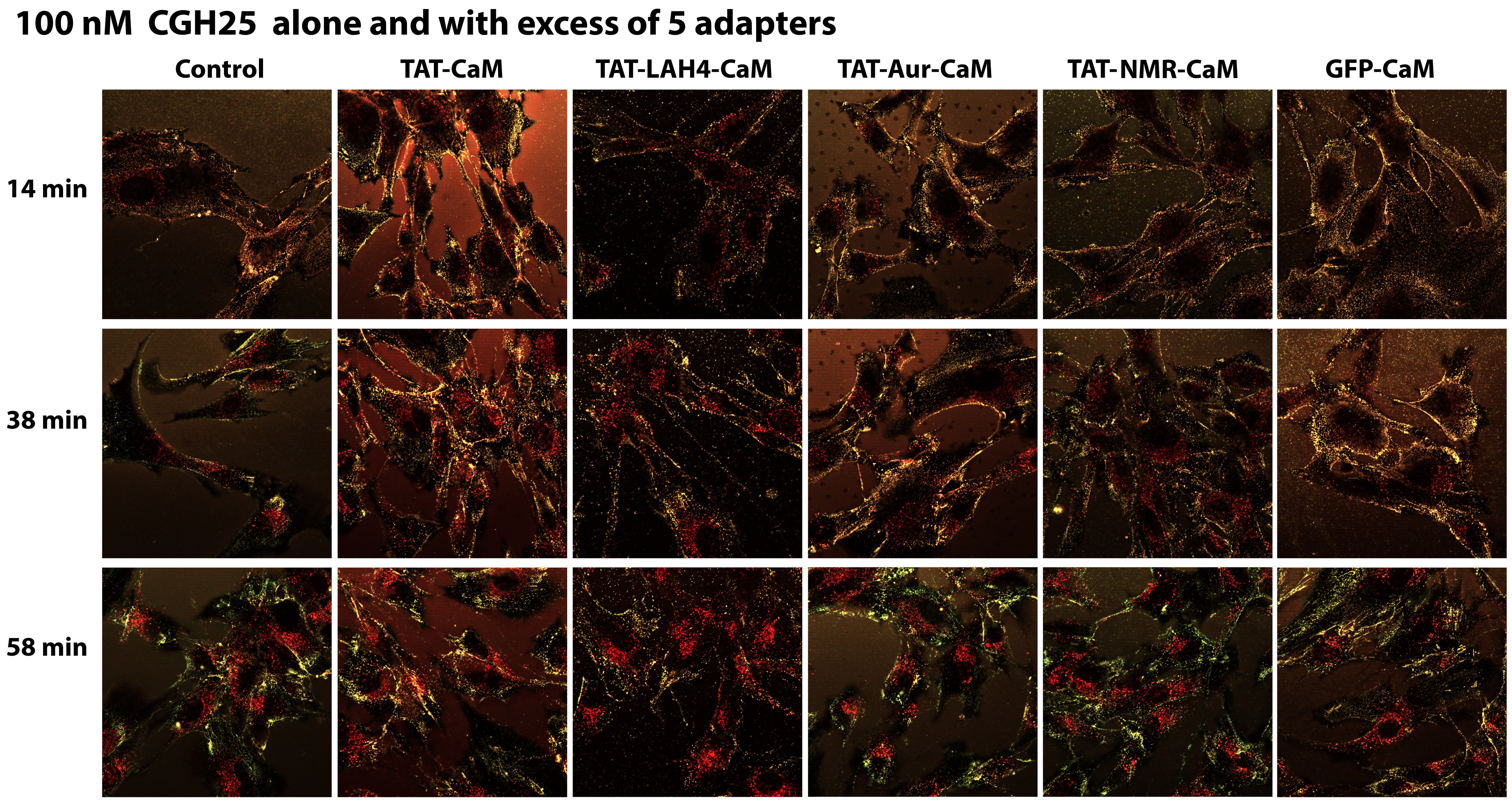
100 nM CGH25 alone and with excess of 5 adapters
Control
TAT-CaM
TAT-LAH4-CaM
TAT-Aur-CaM
TAT-NMR-CaM
GFP-CaM
14 min
38 min
58 min
