# Supplementary material for: Utilization of cell-penetrating peptide adaptors to enhance delivery of variably charged protein cargos
Source: PLoS One. 2026 Jul 10;21(7):e0345530. doi: 10.1371/journal.pone.0345530 (PMC13354093; doi:10.1371/journal.pone.0345530)

# 100 nM CGH36 alone and with excess of 5 adapters

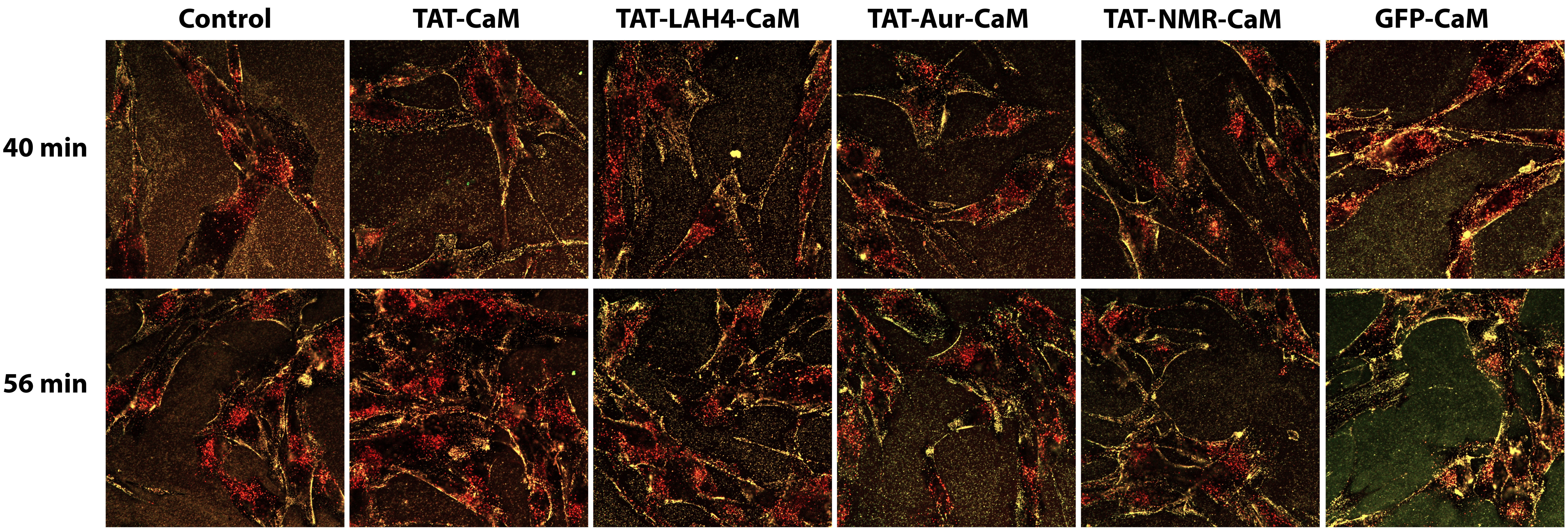

Supplement: S12 Fig — Internalization of 100 nM CGH36 alone or with 500 nM TAT-CaM, TAT-LAH4-CaM and TAT-AUR-CaM or 110nM TAT-NMR-CaM and GFP-CaM. Representative experiment showing profiles of cargo internalization imaged beginning 40 and 56 min after complex addition (56 min used in Fig 6E). (n = 3). (PDF) [file pone.0345530.s012.pdf]
